# Fecal microbiota transplantation in obesity: a comprehensive overview from basic research to clinical application

**DOI:** 10.3389/fmicb.2026.1840517

**Published:** 2026-06-10

**Authors:** Yuxiu Ji, Lijia Zhao, Li Wang, Aiyuan Wang, Chi Zhang, Yujie Xie, Xi Luo

**Affiliations:** 1 Department of Rehabilitation Medicine, The Affiliated Hospital of Southwest Medical University, Luzhou, Sichuan, China; 2 Rehabilitation Medicine and Engineering Key Laboratory of Luzhou, Department of Rehabilitation Medicine, Southwest Medical University, Luzhou, Sichuan, China; 3 Department of Rehabilitation Medicine, Southwest Medical University, Luzhou, Sichuan, China

**Keywords:** bibliometric analysis, fecal microbiota transplantation, metabolism, microbiota dysbiosis, obesity, overweight

## Abstract

**Background:**

Obesity involves microbiota dysbiosis, low-grade inflammation, and insulin resistance, which interacts with multiple metabolic disorders. Fecal microbiota transplantation (FMT) is an emerging therapeutic approach in obesity that enhances intestinal barrier function and regulates energy metabolism.

**Aim:**

To provide a comprehensive overview of publication trends, research collaborations, hotspots, future directions, and the current clinical application status of FMT in obesity.

**Methods:**

Literature searches were conducted in the Web of Science (WoS) and PubMed databases. The primary analysis was performed using the WoS database for bibliometric analysis, while PubMed was searched to supplement the clinical research landscape, ensuring data comprehensiveness and methodological rigor.

**Results:**

A total of 517 papers were finally included, of which 116 (22.44%) were published in the top 10 academic journals. Most publications originated from China (*n* = 246, 47.6%) and Zhejiang University contributed the most publications (*n* = 18, 3.5%). *Gut Microbes* ranked first (21 publications, IF 10.931), followed by *Frontiers in Microbiology* (19, IF 4.504) and *Nutrients* (14, IF 4.919). Research hotspots have shifted toward “targeting the gut microbiota.” “Oral supplementation” and targeted “prebiotics” may be more accessible in the future. The mini literature review of 21 clinical trials revealed that metabolic improvements following FMT are often transient and highly variable across individuals. No standardized protocol for donor selection, delivery route, or outcome measurement currently exists, and most trials had small sample sizes.

**Conclusion:**

Although FMT shows promise, its clinical benefits remain transient and variable across individuals. Current evidence does not yet support routine clinical application. Targeted oral microbiota supplementation may represent a future direction, but high-quality, large-scale clinical trials are urgently needed to establish standardized protocols and evaluate long-term safety and efficacy.

## 1 Introduction

Obesity is a global epidemic characterized by chronic low-grade inflammation and insulin resistance, driven in part by gut microbiota dysbiosis (Apovian, 2016; Endalifer and Diress, 2020; Ncd Risk Factor Collaboration (Ncd-RisC), 2024), leading to chronic musculoskeletal disorders, metabolic load, acute myocardial infarction, and premature death (Endalifer and Diress, 2020). Timely intervention and management of obesity are mandatory. Conventional lifestyle interventions based on dietary manipulation and physical exercise are effective but hard-to-sustain. Microbiota-targeted therapy, especially fecal microbiota transplantation (FMT), has emerged as a novel strategy to restore microbial homeostasis and energy regulation (König et al., 2017).

Concerns about FMT safety and efficacy have spurred increasing research into its role in weight management. Early research primarily focused on mechanisms underlying FMT in obesity regulation and the identification of core “obesity-related microbiome” sequences in animal models. With the progressive application of FMT in clinical diseases, recent studies have shifted toward metabolic improvements and microbial changes in patients. While animal studies consistently demonstrate metabolic benefits of FMT in obesity, clinical trial results remain discordant. The overall therapeutic efficacy is not yet well established, and substantial uncertainty persists regarding donor selection, delivery protocols, and long-term safety. Few studies have systematically mapped the research landscape or addressed these translational gaps (Zhang et al., 2025).

To address this, we conducted a bibliometric analysis combined with a mini-review of relevant literature retrieved from the Web of Science (WoS) database and PubMed. By synthesizing the characteristics of the retrieved literature, we analyzed publication trends, collaboration networks, research hotspots, and current clinical evidence. This study aims to offer a comprehensive perspective to inform future research directions.

## 2 Materials and methods

### 2.1 Data sources and search strategy

We employed a multi-database strategy to investigate the research landscape and identify potential hotspots, rather than merging databases, due to differences in methodology and data compatibility. The primary analysis was conducted using the Web of Science Core Collection (WoSCC), which provides standardized citation indexing and seamless compatibility with widely used bibliometric tools such as CiteSpace and VOSviewer, thereby ensuring analytical reliability and efficiency (Ding and Yang, 2022). PubMed was searched separately as a supplementary analysis to ensure robustness of data sources; it was selected for retrieving clinical trials owing to its standardized indexing and focus on biomedical research (Ossom Williamson and Minter, 2019). Given the substantial overlap in literature coverage, we ultimately used the WoSCC database for bibliometric analysis and the PubMed database for a mini-review of clinical research progress (e.g., FMT types, pre-FMT preparation, and delivery methods). This tiered strategy balances comprehensive analysis depth with cross-database validation breadth.

The literature search for bibliometric analysis was performed in the WoSCC, including the Science Citation Index (SCI) and Social Science Citation Index (SSCI), covering the period from inception to August 20, 2025. The search query consisted of two components: FMT (search term #1: TS = (“fecal microbiota transplantation”)) and obesity (search term #2: TS = (“obesity”)). Detailed searching information is provided in the Supplementary Table 1. Publication types were restricted to “Article” and “Review Article” published in English. A summary of the data source and selection process is presented in Supplementary Table 2. For clinical evidence and efficacy analysis, we further searched for clinical trials in the PubMed database on August 30, 2025, without publication year restriction (i.e., from database inception, 1996, to August 30, 2025), using a search query consistent with that used in WoSCC, and limited the article type to “Clinical Trial” (Supplementary Table 3).

### 2.2 Literature screening and data extraction

**FIGURE 1:**
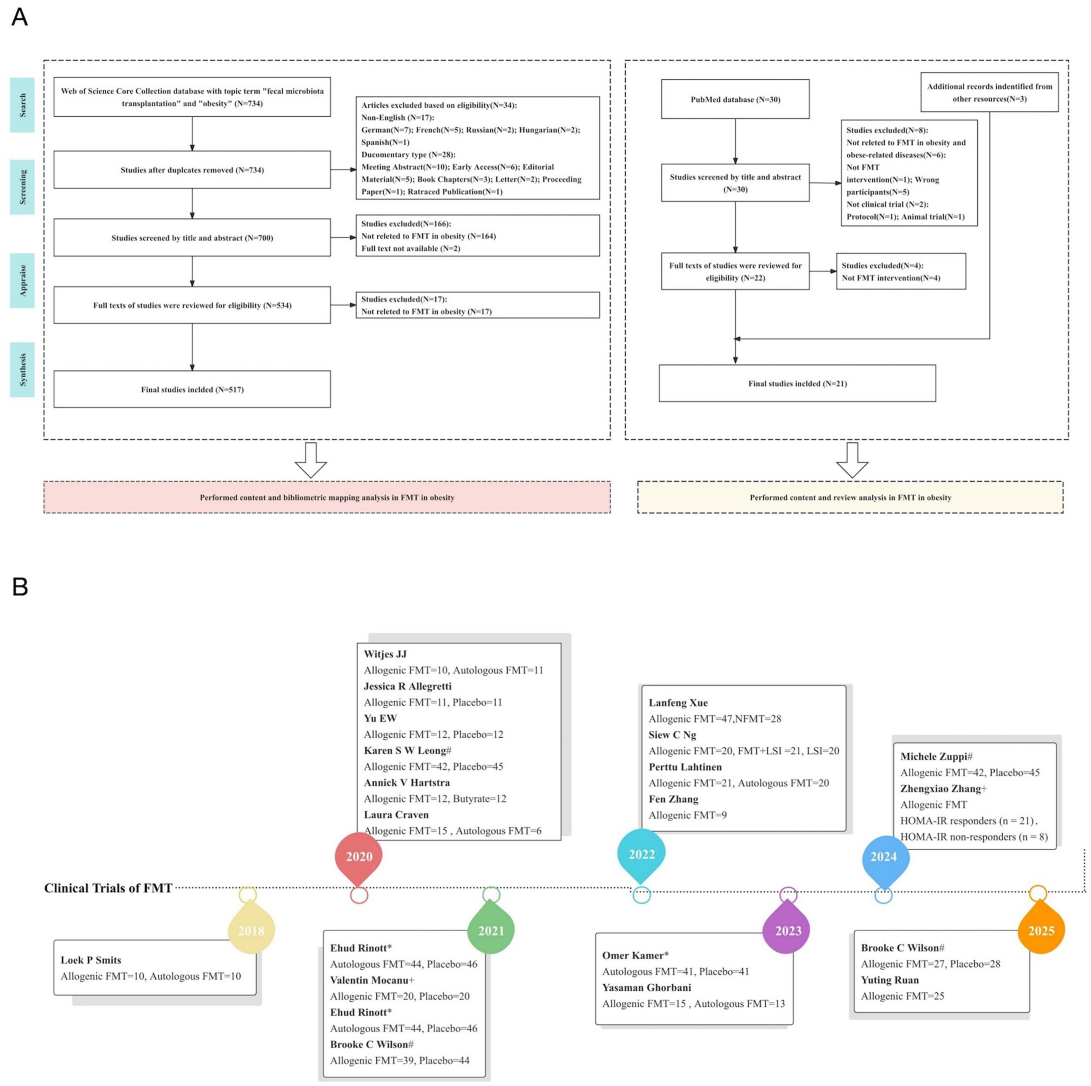
The overall of included literatures. **(A)** Literature screening flow chat on WoS and PubMed. **(B)** The overall of clinical trials from PubMed (The same trial register number: ^+^: NCT02741518, *: NCT03477916, ^#^: ACTRN12615001351505).

A systematic literature search and data extraction from the WoSCC database were conducted independently by two researchers (LJZ and LW). Relevant information, including titles, abstracts, keywords, authors, and references, was downloaded in TXT format. Subsequently, AYW and XL independently cross-checked and excluded articles according to the predefined criteria. Any disagreements during the selection process were resolved through discussion with a third party (CZ and YJX). Following a rigorous screening and appraisal process, 517 papers were included for bibliometric analysis (Figure 1A). Similarly, two independent authors (LJZ and LW) initially identified 30 clinical trials on FMT in obesity in PubMed, with no publication year restriction. An additional three records were included after screening references and related articles. Ultimately, 21 clinical trials met the inclusion criteria and were included in the review analysis (Figure 1A). An overview of the trial characteristics is presented in Figure 1B.

### 2.3 Bibliometric analysis

Bibliometric analysis quantitatively reviews and describes published literature, allowing systematic evaluation of a research field. VOSviewer (version 1.6.20) is a software tool designed to construct and visualize bibliometric networks and extract key information from publications (van Eck and Waltman, 2010). It is commonly used to analyze co-occurrence relationships among authors, journals, and keywords (Zeng et al., 2024). In this study, we conducted a co-authorship analysis of countries, institutions, authors, and journals to reveal collaborative networks. In addition, we performed co-citation analysis of authors, journals, and references to identify key contributions in the field of microbiota and obesity. Co-citation is a metric used to assess the relationship between two documents based on their joint citation by a third document. The more citations a document receives, the greater its perceived importance within a given field. Citation analysis thus serves to identify the intellectual foundation of a research field, with the most highly cited works representing the foundational knowledge and primary focus of the scientific community (Zeng et al., 2024). All quantitative data were exported in TXT format and processed using Microsoft Office Excel 2023. CiteSpace (version 6.2.R4) supports the exploration of secondary data extracted from various online databases through visualized knowledge mapping (Chen, 2004; Synnestvedt et al., 2005). Here, we used CiteSpace to visualize research hotspots and frontiers by analyzing keywords using timezone and timeline views in the context of FMT and obesity (Mirakyan, 2021). Furthermore, we performed burst detection analysis for keywords and references to capture sudden changes in research trends over specific periods (Chen et al., 2013). Parameter settings for CiteSpace are indicated in the corresponding figures.

Bibliometric analyses are sensitive to keyword heterogeneity, including synonym variants, singular/plural forms, acronyms, and spelling differences. To minimize this variability and ensure accurate co-occurrence and burst detection, we performed a systematic keyword normalization procedure. First, author keywords and Keywords Plus (from WoSCC) were extracted and merged. Second, two independent reviewers (LJZ and LW) manually reviewed all unique keyword entries and unified synonymous terms based on the MeSH (Medical Subject Headings) thesaurus and consensus discussions. Third, plural forms were converted to singular where semantically equivalent. Fourth, general or non-informative terms were excluded from co-occurrence analysis. Finally, the cleaned keyword set was used for all subsequent analyses, including co-occurrence mapping, cluster analysis, and burst detection.

## 3 Results

### 3.1 Overall research output and growth trend

**FIGURE 2:**
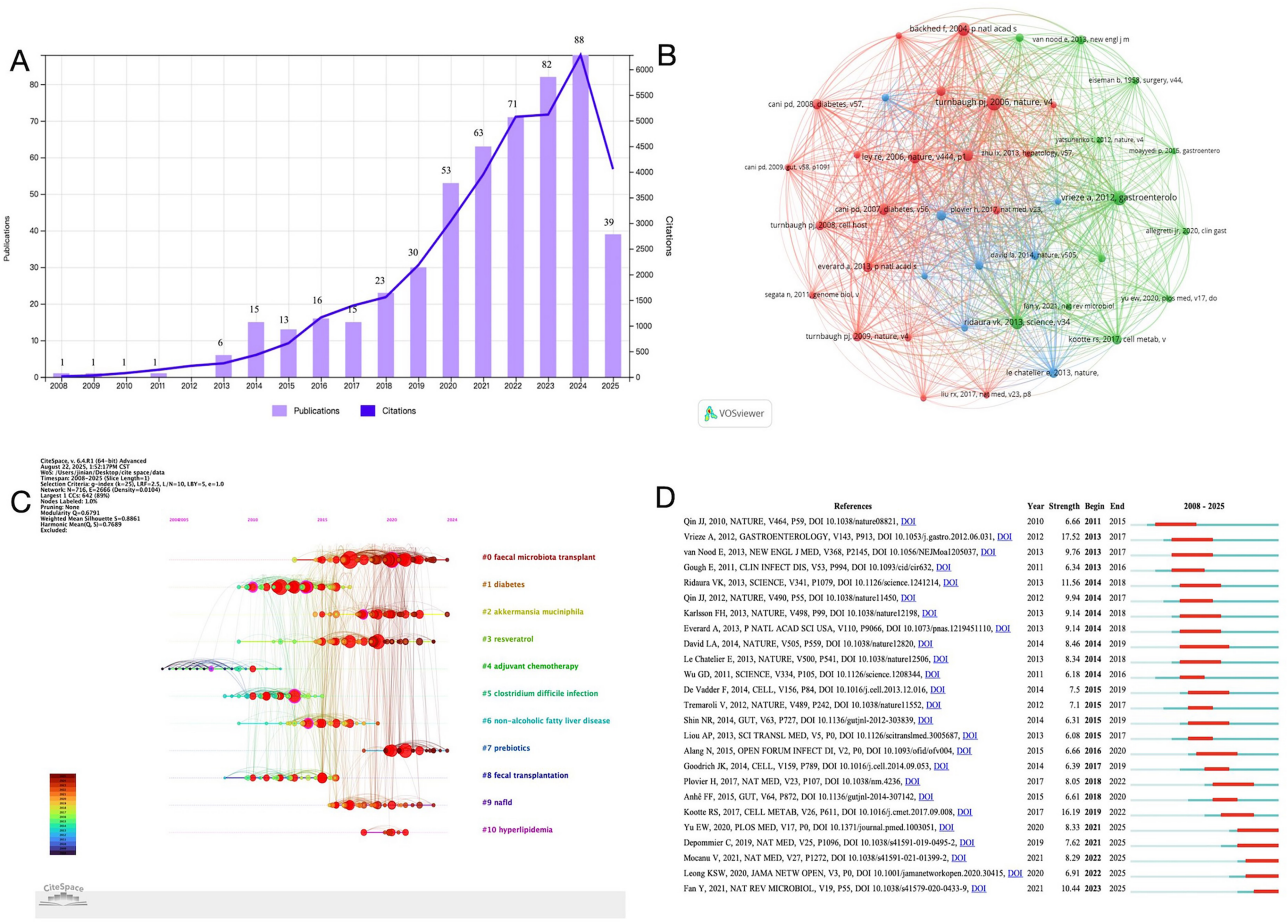
The overall research output. **(A)** Growth trend of publications and citation of FMT in obesity. **(B)** The visualization of co-cited references. **(C)** Timeline view illustrates the independent evolution and interactions among clusters over time. In timeline diagram, each horizontal line represents a cluster sorted from top to bottom according to the size (#0 is usually the largest cluster). The vertical axis represents time span showing the scope of the study. **(D)** Top 25 references with the strongest citation bursts involved of FMT in obesity. The red bars indicate citation burstiness. Burst keywords indicate the research frontier in this field.

A total of 517 papers were published by 3,550 authors from 979 organizations in 58 countries, published in 250 journals, and citing 27,531 references from 3,754 journals (Figure 1A). The annual publications related to FMT in obesity has shown a rising trend, reflecting sustained and vigorous attention in this field. From 2008 to 2025, 88 articles with 6,280 citations were published in 2024, ranking first in recent decades and indicating a continued research hotspot in the future (Figure 2A).

A total of 27,531 co-cited references related to FMT in obesity were identified, and the top 10 co-cited references elucidate the research basis of gut microbiota therapy in obesity and obesity-related diseases (Bäckhed et al., 2004; Ley et al., 2005, 2006; Turnbaugh et al., 2006; Cani et al., 2007, 2008; Qin et al., 2012; Vrieze et al., 2012; Everard et al., 2013; Ridaura et al., 2013). These highly co-cited references illustrate the evolution of FMT in obesity treatment–from observing microbial phenomena to analyzing mechanisms, and subsequently to clinical intervention–spanning the period from 2004 to 2013 (Table 1). Half of the highly co-cited articles were published between 2010 and 2015, indicating that the core directions and foundational knowledge were established before 2015. A total of 40 references with at least 32 co-citations were included in the co-citation network, which was divided into three clusters, corresponding to the three colors displayed by VOSviewer (Figure 2B). “Turnbaugh PJ, 2006, Nature” exhibits active co-citation relationships with “Vrieze A, 2012, Gastroenterology,” “Ridaura VK, 2013, Science,” “Bäckhed F, 2004, P Natl ACAD Sci USA,” among others. The closest clusters were “#0 fecal microbiota transplant,” “#1 diabetes,” “#2 *Akkermansia muciniphila* (*A. muciniphila*),” “#3 resveratrol,” and “#7 prebiotics” in timeline visualization (Figure 2C). The top 25 references with the strongest citation bursts are presented in Figure 2D, with burst strengths ranging from 6.08 to 17.52 and durations spanning 2–5 years. A detailed summary of the research content is provided in Table 2 (Qin et al., 2010, 2012; Gough et al., 2011, van Nood et al., 2013; Le Chatelier et al., 2013; Wu et al., 2011; Tremaroli and Bäckhed, 2012; Vrieze et al., 2012; Everard et al., 2013; Liou et al., 2013; Karlsson et al., 2013; Ridaura et al., 2013; De Vadder et al., 2014; Goodrich et al., 2014; David et al., 2014; Shin et al., 2014; Anhê et al., 2015; Alang and Kelly, 2015; Plovier et al., 2017; Kootte et al., 2017; Depommier et al., 2019; Yu et al., 2020; Leong et al., 2020; Mocanu et al., 2021; Fan and Pedersen, 2021). The initial citation bursts occurred in 2011, while the most recent was observed in 2025. The reference with the strongest citation burst (strength = 17.52) ranked second in Figure 2D and is titled “Transfer of Intestinal Microbiota From Lean Donors Increases Insulin Sensitivity in Individuals With Metabolic Syndrome,” by Vrieze A with citation bursts from 2013 to 2017 (Vrieze et al., 2012).

**TABLE 1:** Top 10 co-cited references involved of FMT in obesity.

Rank	Co-cited reference	Main research finding	Counts
1	Turnbaugh PJ, 2006, Nature, V444, P1027 (Turnbaugh et al., 2006)	A study found that the abundance of the two dominant bacterial groups in the gut of individuals with obesity correlates with weight loss.	184
2	Vrieze A, 2012, Gastroenterology, V143, P913 (Vrieze et al., 2012)	A study investigated the effects of infusing intestinal microbiota from lean donors into recipients with metabolic syndrome on microbiota composition and glucose metabolism.	146
3	Ridaura VK, 2013, Science, V341, P1079 (Ridaura et al., 2013)	A study analyzed the effects of diet–microbiota interactions using fecal microbiota transplantation.	133
4	Bäckhed F, 2004, P Natl ACAD Sci USA, V101, P15718 (Bäckhed et al., 2004)	Gut microbiota is an important environmental factor that affects energy harvest from diet and energy storage in the host.	121
5	Ley RE, 2006, Nature, V444, P1022 (Ley et al., 2006)	A study indicated that obesity has a microbial component, involving alterations in the abundance of Bacteroidetes and Firmicutes.	102
6	Cani PD, 2007, Diabetes, V56, P1761 (Cani et al., 2007)	A study found that the LPS/CD14 system modulates insulin sensitivity and influences the onset of diabetes and obesity.	89
7	Ley RE 2005, P Natl ACAD Sci USA, V102, P11070 (Ley et al., 2005)	A study analyzed 5,088 bacterial 16S rRNA gene sequences from the microbiota of genetically obese and lean mice.	88
8	Everard A, 2013, P Natl ACAD Sci USA, V110, P9066 (Everard et al., 2013)	A study analyzed the effects of *Akkermansia muciniphila* in obesity and metabolic disorders, providing substantial insight into the underlying mechanisms.	80
9	Cani PD, 2008, Diabetes, V57, P1470 (Cani et al., 2008)	Changes in gut microbiota control metabolic endotoxemia, inflammation, and associated disorders through a mechanism that may increase intestinal permeability.	76
10	Qin JJ, 2012, Nature, V490, P55 (Qin et al., 2012)	A study developed a protocol for a metagenome-wide association study (MGWAS) and compared the genetic content of the fecal microbiota between healthy individuals and those with diabetes.	76

**TABLE 2:** The main research contents of the 25 references with strong citations bursts.

Rank	Strength	Main research finding
1	6.66	A study described the Illumina-based metagenomic sequencing, assembly, and characterization of 3.3 million non-redundant microbial genes derived from 576.7 gigabases of sequence obtained from fecal samples of 124 European individuals (Qin et al., 2010).
2	17.52	A study investigated the effects of infusing intestinal microbiota from lean donors into recipients with metabolic syndrome on microbiota composition and glucose metabolism (Vrieze et al., 2012).
3	9.76	Infusion of donor feces was significantly more effective for the treatment of recurrent *Clostridium difficile* infection than vancomycin (van Nood et al., 2013).
4	6.34	A systematic literature review examined intestinal microbiota transplantation for recurrent *Clostridium difficile* infection and pseudomembranous colitis (Gough et al., 2011).
5	11.56	A study analyzed the effects of diet–microbiota interactions using fecal microbiota transplantation (Ridaura et al., 2013).
6	9.94	A study developed a protocol for a metagenome-wide association study (MGWAS) and compared the genetic content of the fecal microbiota between healthy individuals and those with diabetes (Qin et al., 2012).
7	9.14	A study characterized the fecal metagenome using shotgun sequencing and developed a mathematical model based on metagenomic profiles that identified type 2 diabetes with high accuracy (Karlsson et al., 2013).
8	9.14	A study analyzed the effects of *Akkermansia muciniphila* in obesity and metabolic disorders, providing substantial insight into the underlying mechanisms (Everard et al., 2013).
9	8.46	Short-term consumption of diets composed entirely of animal or plant products alters microbial community structure and overwhelms inter-individual differences in microbial gene expression (David et al., 2014).
10	8.34	An article reported the human gut microbial composition in a population sample of 123 non-obese and 169 obese Danish individuals (Le Chatelier et al., 2013).
11	6.18	Diet strongly affects human health, partly by modulating gut microbiome composition; alternative enterotype states are associated with long-term diet (Wu et al., 2011).
12	7.5	A study demonstrated that the short-chain fatty acids propionate and butyrate, generated by fermentation of soluble fiber by the gut microbiota, activate intestinal gluconeogenesis via complementary mechanisms (De Vadder et al., 2014).
13	7.1	A study reviewed the mechanisms and interactions between the human gut microbiota and obesity, cardiovascular disease, and metabolic syndrome (Tremaroli and Bäckhed, 2012).
14	6.31	Modulation of the gut microbiota (e.g., through an increase in *Akkermansia* spp.) may contribute to the antidiabetic effects of metformin, suggesting that pharmacological manipulation of the gut microbiota could represent a potential treatment for type 2 diabetes (Shin et al., 2014).
15	6.08	A study provided the first empirical support for the claim that changes in the gut microbiota contribute to reduced host weight and adiposity after Roux-en-Y gastric bypass surgery (Liou et al., 2013).
16	6.66	A study reported a case of a woman successfully treated with fecal microbiota transplantation who developed new-onset obesity after receiving stool from a healthy but overweight donor (Alang and Kelly, 2015).
17	6.39	A study compared microbiota samples and found an association between Christensenellaceae and low body mass index, indicating that host genetics influence the composition of the human gut microbiome and may impact host metabolism (Goodrich et al., 2014).
18	8.05	A study analyzed the mechanisms by which *Akkermansia muciniphila* prevents obesity (Plovier et al., 2017).
19	6.61	A study investigated the metabolic impact of a cranberry extract in high-fat, high-sucrose-fed mice and determined that its consequent antidiabetic effects are related to *Akkermansia muciniphila* (Anhê et al., 2015).
20	16.19	A study investigated the role of intestinal microbiota in insulin resistance and found beneficial effects of lean-donor fecal microbiota transplantation on glucose metabolism (Kootte et al., 2017).
21	8.33	A study evaluated the safety of weekly oral fecal microbiota transplantation capsules and their ability to alter gut microbiota and improve metabolic outcomes in patients with obesity (Yu et al., 2020).
22	6.62	A study provided evidence for the safety and tolerability of daily oral supplementation with *Akkermansia muciniphila* bacteria in overweight and obese insulin-resistant volunteers (Depommier et al., 2019).
23	8.29	A study provided proof of concept for the use of a single-dose oral fecal microbiota transplantation combined with daily low-fermentable fiber supplementation to improve insulin sensitivity in patients with severe obesity and metabolic syndrome (Mocanu et al., 2021).
24	6.91	A study assessed the efficacy of fecal microbiota transplantation for treating adolescent obesity and improving metabolism (Leong et al., 2020).
25	10.44	A study discussed current knowledge on how the gut microbiota may link to the pathogenesis of common metabolic diseases, highlighted examples of microbiota-targeted interventions aimed at optimizing metabolic health, and provided future perspectives (Fan and Pedersen, 2021).

Rank: order of references by citation burst strength (highest first). Strength: a quantitative measure of the intensity of citation burst; higher values indicate more rapid and significant increase in citation frequency during the burst period.

### 3.2 Research cooperation status analysis

#### 3.2.1 Countries and institutions analysis

**FIGURE 3:**
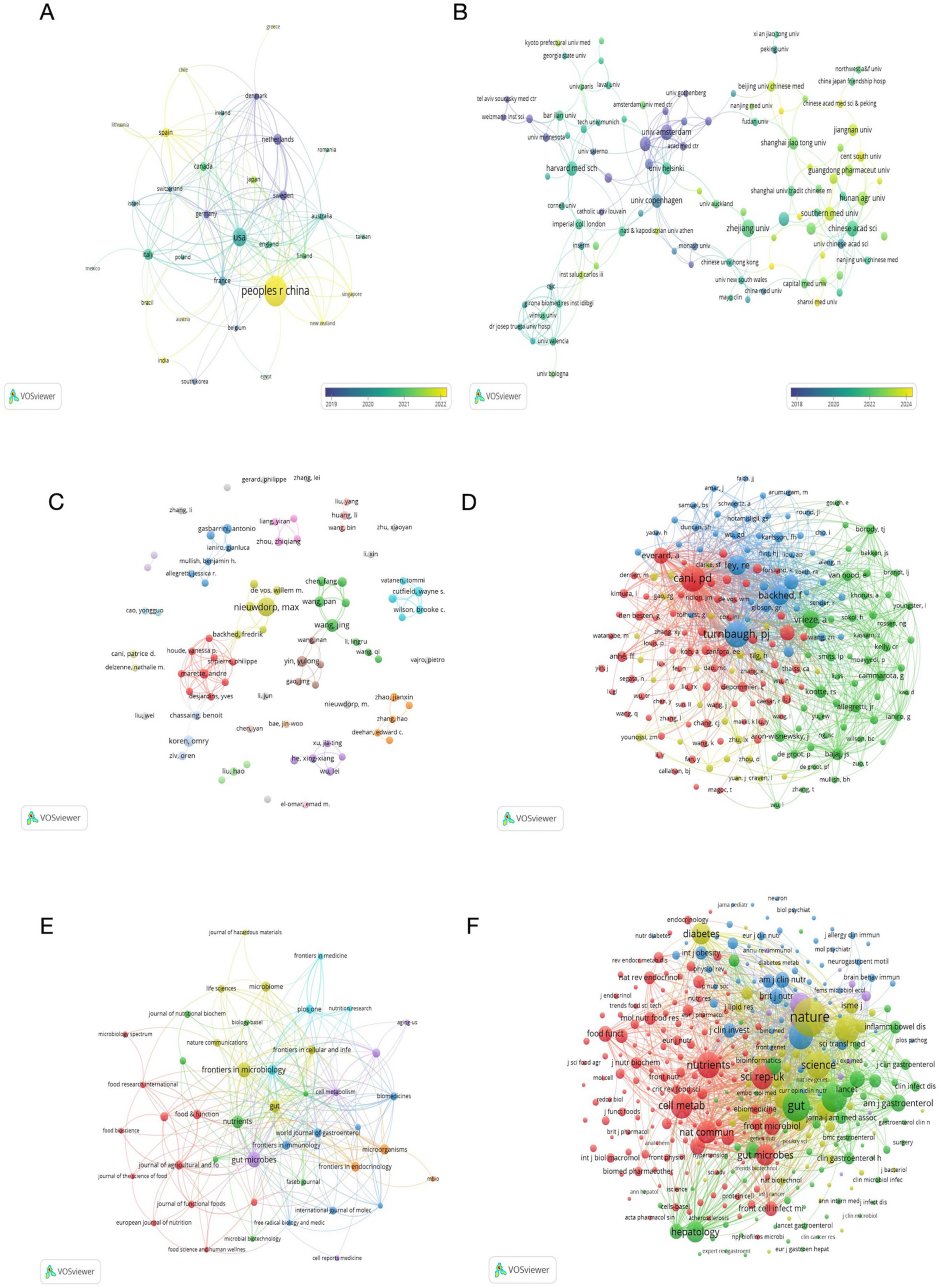
Research cooperation status analysis. The visualization map of countries **(A)**, institutions **(B)**, authors **(C)**, co-cited authors **(D)**, journals **(E)** and co-cited journals **(F)**. The nodes represent co-cited countries/institutions/authors/journals. The higher the number of co-citations or co-occurrences, the larger the nodes. The links between nodes represent co-citation or cooperation relationships. The higher the frequency of co-citation/cooperation, the thicker the links.

These publications originated from 58 countries and 979 institutions. Among the top 10 most productive countries, half were in Europe (*n* = 5) (Supplementary Table 4). China contributed the largest number of publications (*n* = 246, 47.6%), accounting for nearly half of the total, following by the United States (USA) (*n* = 93, 18.0%). Figure 3A presents the collaboration network of the involved countries based on publication volume and relationships, with node colors indicating the year of highest publication output (ranging from 2019 to 2022). The USA demonstrated the most extensive and active collaborations with England, Australia, France, Canada, and Italy. Early collaboration, shown in green, was primarily between the USA, England, and other European countries, whereas more recent collaboration (in yellow) has shifted to involve East Asian countries alongside several European nations, such as Sweden, Spain, and the Netherlands.

China also dominated the list of top 10 institutions, accounting for 60% of them, consistent with its national publication output (Supplementary Table 4). The top three institutions were Zhejiang University (*n* = 18, 3.5%), Chinese Academy of Sciences (*n* = 15, 3.0%), and University of Amsterdam (*n* = 15, 3.0%). As shown in the Figure 3B, the collaborative relationship among 979 institutions with at least three publications was more extensive than the country-level network. Zhejiang University, Chinese Academy of Sciences, Harvard Medical School, and the University of Amsterdam exhibited close collaborations with numerous universities and research centers. However, institutional collaboration networks tended to remain domestic or regionally confined; for instance, Chinese universities showed limited collaboration with foreign institutions.

#### 3.2.2 Authors and co-cited authors analysis

More than 3000 authors have contributed to research in the microbiota-obesity field. Among the top 10 most prolific authors, each had published at least five articles, with half affiliated with Chinese institutions, underscoring the leading role of East Asian researchers in this domain. Nieuwdorp M was the most productive author, with 19 publications (Supplementary Table 5). And 82 authors were included in the collaboration network with at least three relevant publications (Figure 3C) by VOSviewer. Collaborative relationships were dispersed across small clusters, distinguished by different colors; for example, the largest cluster (red) reflects positive collaboration among Marette A, Houde V, Desjardins Y, and St-Pierre P, among others.

VOSviewer identified a total of 20,239 co-cited authors. The top 10 co-cited authors, with at least 77 citations, have made substantial contributions to this field (Supplementary Table 5). Four authors were co-cited more than 200 times: Cani PD (*n* = 377), followed by Turnbaugh PJ (*n* = 371), Bäckhed F (*n* = 225), and Ley RE (*n* = 216). A co-citation network map was generated comprising 201 authors with at least 20 co-citations (Figure 3D). Turnbaugh PJ exhibited active co-citation connections with multiple authors, including Bäckhed F, Ley RE, and Qin JJ.

#### 3.2.3 Journals and co-cited journals analysis

A total of 250 academic journals have published articles on FMT in obesity, reflecting strong interest in microbiota-obesity topics within these periodicals. *Gut Microbes* ranked first (*n* = 21, IF = 10.931), followed by *Frontiers in Microbiology* (*n* = 19, IF = 4.504) (Supplementary Table 6). Among all publications, only 116 appeared in the top 10 journals, accounting for 22.44% of the total. Of these top 10 journals, 50% (5/10) were published in Switzerland, and 30% (3/10) in the UK. Among the top 10 journals, *Gut* had the highest impact factor (*n* = 12, IF = 25.503), followed by *Microbiome* (*n* = 9, IF = 12.683). Positive citation relationships among different journals are shown in Figure 3E.

Supplementary Table 6 also presents the top co-cited journals as identified by VOSviewer, all of which had more than 600 citations. *Nature* ranked first with 1,708 co-citations (IF = 47.84), followed by *Gut* (1,136 co-citations, IF = 25.503), *Gastroenterology* (954 co-citations, IF = 25.214), *Proceedings of the National Academy of Sciences of the USA* (933 co-citations, IF = 8.872), and *Science* (885 co-citations, IF = 44.692). Figure 3F presents a co-citation network map of 351 journals, each with at least 20 co-citations; larger nodes represent journals that have published highly influential articles in this field. These findings offer valuable guidance for researchers selecting appropriate journals for future submissions in microbiota-obesity research.

### 3.3 Research hotspots and frontiers

The top 25 high-frequency keywords identified by VOSviewer are summarized in Table 3. Notably, the occurrence of “microbiota” (counts = 64) and its variant forms accounted for 24% (6/25) of these terms, including “gut microbiota” (counts = 278), “intestinal microbiota” (counts = 146), “microbiome” (counts = 45) and “gut microbiome” (counts = 65). The terms “microbiome” and “microbiota” are often used interchangeably; however, strictly speaking, *microbiota* refers to the microbial taxa associated with complex organisms such as humans, whereas *microbiome* refers to the catalog of these microbes and their genes (Mohajeri et al., 2018). With advances in gene sequencing technologies, studies have revealed that obesity is closely associated with gut dysbiosis, which can produce active signaling molecules that interact with host metabolism. For instance, short-chain fatty acids (SCFAs) influence insulin sensitivity and regulate energy metabolism in the host (Nicholson and Wilson, 2003; Bäckhed et al., 2004; Nicholson et al., 2005; Neves et al., 2015). Obesity is characterized by oxidative stress and low-grade inflammation, increasing the risk of type 2 diabetes mellitus (T2DM), dyslipidemia, and hypertension (Boulangé et al., 2016). The high frequency of keywords such as “inflammation” (counts = 146), “insulin-resistance” (counts = 76), “insulin sensitivity” (counts = 65), and chain fatty acids (counts = 58) indicates a shift from associative studies toward mechanistic investigations.

**TABLE 3:** Top 25 keywords on research of application of FMT in obesity.

Rank	Keywords	Counts	Rank	Keywords	Counts
1	Obesity	320	14	Diet	57
2	Gut microbiota	278	15	Health	56
3	Intestinal microbiota	146	16	Metabolism	52
4	Fecal microbiota transplantation	131	17	Microbiome	45
5	Inflammation	104	18	*Akkermansia muciniphila*	42
6	Metabolic syndrome	89	19	Dysbiosis	41
7	Insulin-resistance	76	20	*Clostridium difficile* infection	40
8	Probiotics	66	21	Mice	40
9	Gut microbiome	65	22	Disease	35
10	Insulin sensitivity	65	23	High-fat diet	35
11	Diet-induced obesity	64	24	Double-blind	32
12	Microbiota	64	25	Impact	30
13	Chain fatty acids	58			

**FIGURE 4:**
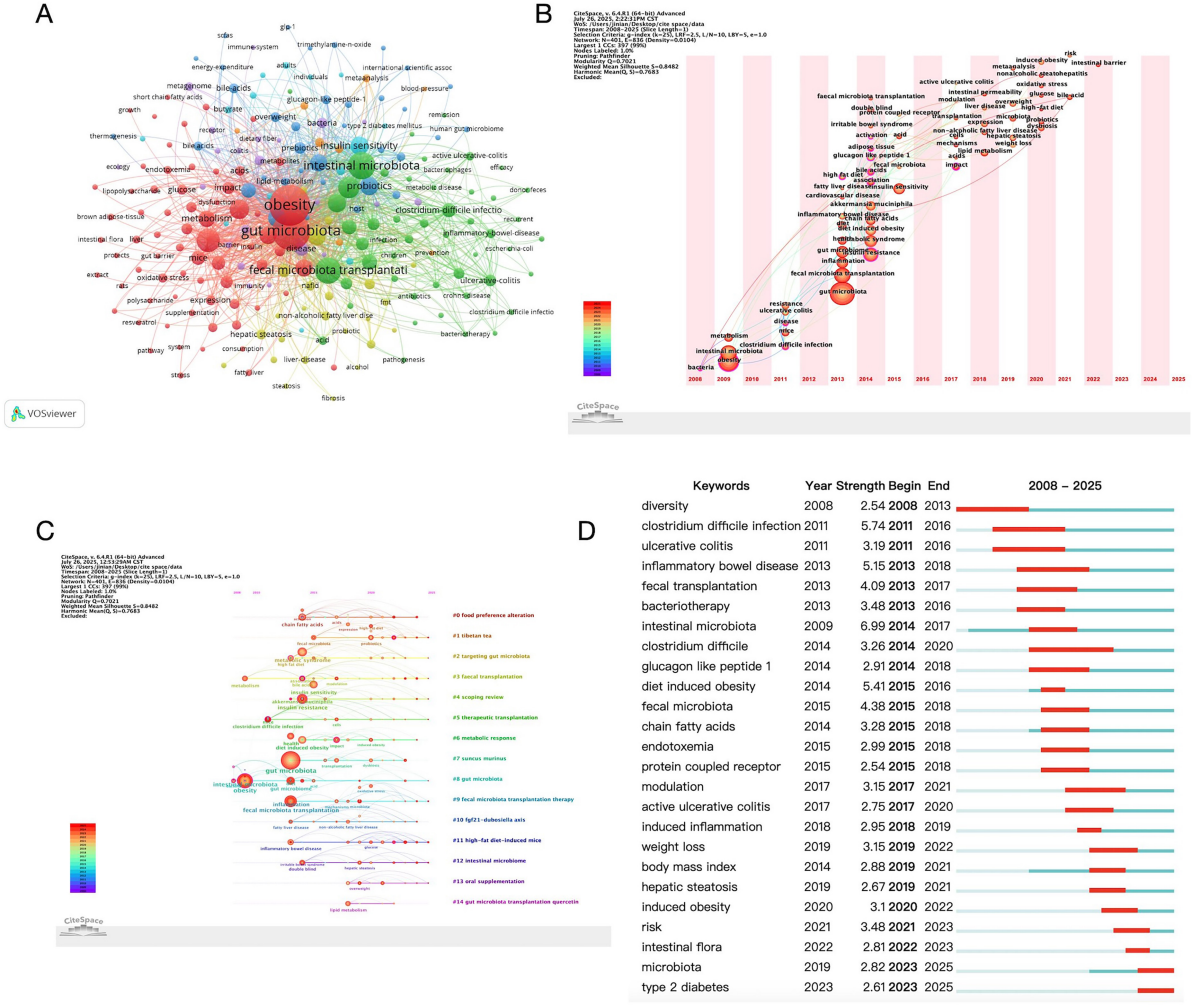
Research hotspots and frontiers analysis. **(A)** The visualization map of keyword co-occurrence analysis. The nodes represent keywords and node size represents keyword frequency. The higher the number of co-occurrences, the larger the nodes. The links between nodes represent co-citation relationships. The higher the frequency of co-citation, the thicker the links. **(B)** Timezone view shows the global time flow, and the importance of nodes was summarized in terms of citation-based metrics. In the timezone view, the horizontal axis represents years, reflecting the changes of the research topic over time. Nodes are placed into the corresponding time zone according to the time of their first occurrence or emergence. The vertical axis represents the topic clusters and the nodes within the same cluster are closer in the vertical direction. **(C)** Timeline view illustrates the independent evolution and interactions among clusters over time. In timeline diagram, each horizontal line represents a cluster sorted from top to bottom according to the size (#0 is usually the largest cluster). The vertical axis represents time span showing the scope of the study. **(D)** Top 25 the strongest citation bursts of keyword cluster. The red bars indicate citation burstiness. Burst keywords indicate the research frontier in this field.

Author keywords with more than five occurrences were included in the co-occurrence bibliometric map generated by VOSviewer (Figure 4A), forming six clusters (red, yellow, blue, green, orange, and purple) representing six distinct research directions. The red cluster primarily focuses on the role of gut microbiota and SCFAs in obesity treatment, investigating associated impacts and mechanisms. The green cluster examines administration routes and the effects of FMT, including the use of probiotics, prebiotics, and oral FMT capsules. Figure 4B illustrates that scholars focused primarily on the association between gut microbiota and metabolic diseases, particularly obesity between 2008 and 2012, with most studies limited to high-fat diet–induced animal models to investigate the effects and mechanisms of FMT in obesity. Since 2013, microbiota-based therapies have entered an era of diversified development, with increasing attention directed toward obesity-related disorders such as fatty liver disease, cardiovascular disease, T2DM, and inflammatory bowel disease.

The keyword co-occurrence network comprised 15 clusters (Figure 4C), and the 15 largest clusters are summarized in Table 4. The most frequent cluster was “food preference alteration” (cluster 0) encompassing terms such as chain fatty acids, acids, expression, and activation. Other clusters covered topics including gut microbiota, therapeutic transplantation, fecal transplantation, and targeting gut microbiota. Detailed keyword information within each cluster is provided in Supplementary Table 7.

**TABLE 4:** The detailed information of the largest 15 clusters.

Cluster ID	Cluster term	Size	Silhouette	Average year	The most cited members
#0	Food preference alteration	14	0.863	2018	Chain fatty acids; acids; expression; activation; fecal microbiota transplantation
#8	Gut microbiota	14	0.884	2013	Obesity; intestinal microbiota; gut microbiome; diet; bacteria
#5	Therapeutic transplantation	13	0.854	2016	Health; *Clostridium difficile* infection; mice; disease; ulcerative colitis
#3	Fecal transplantation	12	0.919	2017	Metabolism; bile acids; association; adipose tissue; modulation
#9	Fecal microbiota transplantation therapy	12	0.851	2019	Fecal microbiota transplantation; inflammation; mechanisms; microbiota; oxidative stress
#2	Targeting gut microbiota	11	0.850	2017	Metabolic syndrome; high fat diet; endotoxemia; increases; gut-liver axis
#1	Tibetan tea	10	0.744	2021	Fecal microbiota; probiotics; high-fat diet; homeostasis; short-chain fatty acids
#4	Scoping review	10	0.878	2017	Insulin resistance; insulin sensitivity; *Akkermansia muciniphila*; glucagon like peptide 1; body mass index
#11	High-fat diet-induced mice	10	0.768	2021	Inflammatory bowel disease; glucose; intestinal permeability; intestinal barrier; bile acid
#6	Metabolic response	9	0.818	2017	Diet induced obesity; impact; induced obesity; liver disease; weight gain
#10	FGF21-*Dubosiella* axis	9	0.932	2018	Fatty liver disease; non-alcoholic fatty liver disease; cardiovascular disease; active ulcerative colitis; consumption
#12	Intestinal microbiome	9	0.831	2020	Double blind; hepatic steatosis; irritable bowel syndrome; nonalcoholic steatohepatitis; steatohepatitis
#7	*Suncus murinus*	8	0.800	2019	Gut microbiota; transplantation; dysbiosis; crohns disease; fecal microbiota transplantation (FMT)
#13	Oral supplementation	7	0.854	2020	Overweight; risk; weight loss; adults; regulatory T cells
#14	Gut microbiota transplantation quercetin	4	1.000	2019	Lipid metabolism; atherosclerosis; intramuscular fat; gut microbiota transplantation; caloric restriction

Silhouette (S) value: a measure of cluster homogeneity, ranging from −1 to 1. Values > 0.7 indicate high internal consistency and well-defined clusters.

We analyzed data from 2008 to 2025 to identify burst keywords, which reflect the evolving trajectory of FMT in obesity treatment. The top 25 burst keywords are presented in Figure 4D. The top 10 keywords by burst strength were as follows: intestinal microbiota (burst strength = 6.99), *Clostridium difficile* infection (5.74), diet induced obesity (5.41), inflammatory bowel disease (5.15), fecal microbiota (4.38), fecal transplantation (4.09), risk (3.48), bacteriotherapy (3.48), chain fatty acids (3.28), Clostridium difficile (3.26). Notably, “microbiota” and “T2DM” both exhibited burst activity from 2023 to 2025, suggesting an emerging key direction, which aligns with previous research indicating that pharmacological modulation of the gut microbiota may represent a potential therapeutic approach for T2DM (Shin et al., 2014).

## 4 Clinical research progression: a mini literature review

Fecal microbiota transplantation has demonstrated positive effects in animal models, sustaining interest in its potential clinical application for obesity. However, clinical evidence in humans remains debatable, highlighting the need for high-quality clinical validation.

To advance the clinical application of gut microbiome research, the scientific community must first address key questions: what constitutes a “healthy” microbiome (Carlucci et al., 2016), which factors influence gut microbiome composition, how the gut microbiome is associated with obesity, and which bacterial taxa dominate obesity-related changes (McBurney and Cho, 2024). As an untargeted strategy for modulating the gut microbiome, the response to FMT appears transient and variable, suggesting that achieving ideal efficacy may require a greater magnitude of microbial engraftment. Nevertheless, there is currently no indicative evidence confirming whether lean phenotypes are successfully transferred to the recipient site or what proportion of microbiome colonization is required for effectiveness. Additionally, several questions remain to be addressed before FMT can be implemented clinically. To explore these hotspots and controversies, we conducted a mini literature review of clinical trials on FMT in obesity using the PubMed database (Figure 1).

### 4.1 Is FMT effective in individuals with obesity?

Previous studies have found that in recipients with obesity, improvements in insulin resistance and changes in gut microbiome typically return to baseline 12–18 weeks after FMT administration, suggesting that multiple FMT sessions may be required to sustain long-term efficacy (Kootte et al., 2017; Yu et al., 2020). The observation that only a subset of individuals exhibits microbiome alterations may be attributed to the multifactorial nature of obesity and insulin resistance. When defining the efficiency of FMT, we should answer the question that which outcomes can be the “successful” indicators. For most participants, the successful and sustained weight loss may be the first answer. However, few studies have set weight loss as the primary outcome; instead, most have focused on metabolic improvements or microbiome alterations in recipients (Lahtinen et al., 2022). The specific obesity-related microbiota disrupts normal energy metabolic homeostasis by inducing inflammation and modulating central appetite via the microbiota-gut-brain axis (Nóbrega et al., 2025). Emerging studies suggest that lifestyle factors, host dietary habits, microenvironmental characteristics, pre-FMT recipient microbiota composition, donor–recipient complementarity, and FMT strategy may be key determinants. Ruan Y further investigated the differences between responders (defined as those with ≥5% weight loss) and non-responders (those with <5% weight loss) following FMT (Ruan et al., 2025). Response to allogenic FMT was characterized by lower fecal microbial diversity at baseline, particularly reduced abundance of *Eubacterium ventriosum* and *Ruminococcus torques* (Kootte et al., 2017). Greater baseline microbial diversity may indicate enhanced host resistance and resilience, traits that could hinder successful engraftment in microbial therapy. Thus, improving the engraftment effect in FMT treatment may be an important procedure. Several key issues warrant further investigation prior to clinical implementation.

### 4.2 Is antibiotic or bowel preparation necessary?

Based on previous experience with microbiome-based therapies in ulcerative colitis, some researchers have proposed using antibiotics to prevent adverse events (AEs) or bowel preparation to enhance engraftment prior to FMT (Fukuyama et al., 2017). Although most studies have not adopted these measures as mandatory procedures (Supplementary Table 8), whether to apply gut preparation or pretreatment antibiotics remains uncertain, owing to a lack of clear experimental evidence and concerns regarding antibiotic use and ethical issues.

### 4.3 Which is the optimal clinical delivery route?

Gut microbiota engraftment is directly influenced by the delivery modality. Although endoscopic procedures (e.g., colonoscopy 90%, upper gastrointestinal route 80%) are effective, their invasiveness may limit their use for repeated FMT administrations (Kao et al., 2017; Yu et al., 2020). Notably, several trials have chosen duodenal delivery because it does not require bowel preparation (Ng et al., 2022). The engraftment effect of FMT capsules has been proven to a noninferior administration to endoscopic procedures under the same amount of fecal material without serious AEs (Youngster et al., 2016; Kao et al., 2017; Yu et al., 2020). Elaine W found that frozen oral FMT capsules administered weekly for 12 weeks were safe, well-tolerated, and effective in individuals with obesity, without prior antibiotic use or bowel preparation (Yu et al., 2020). Capsule-based fecal microbiota transplantation (cFMT) may be a more popular and accessible delivery method in future clinical application, but the popularization of cFMT need to establish a pharmaceutical-grade standardized preparation and quality supervisory system to trace potential long-term AEs. Moreover, the emergence of cFMT may catalyze the “specific microbiota agents” for targeted diseases or patients (Yu et al., 2025).

### 4.4 Should FMT be combined with lifestyle intervention?

Diet and exercise interact synergistically with the gut microbiota by promoting gut microbiota modulation in enhancing metabolism and maintaining immune homeostasis (Campbell et al., 2016). Siew CN found that FMT combined with lifestyle intervention increased the abundance of *Bifidobacterium* and *Lactobacillus* compared with FMT alone (Ng et al., 2022). However, most clinical trials didn’t include dietary intervention or exercise intervention (Supplementary Table 8). Exercise alters gut microbiota composition and function, but dose-response relationships remain unclear (Kelley et al., 2014; Campbell et al., 2016; Allen et al., 2018; Carbajo-Pescador et al., 2019; Quiroga et al., 2020). Diet shapes microbial engraftment by providing substrates and nutrients required for proliferation and survival (Beam et al., 2021). High-fiber intake may enhance engraftment during FMT by restructuring microbiota composition and improving function through increased nutrient availability for microbial metabolism and growth. In contrast, bile acids (BAs), which exert antimicrobial properties by inducing cathelicidin expression or disrupting microbial cell walls, show an inverse correlation with α−diversity and the engraftment of newly transferred microbes following FMT. To get a better clinical efficacy, standardized FMT procedures should be combined with individualized lifestyle interventions to reinforce metabolic response in future.

### 4.5 Which type of FMT is better?

Among the clinical trials, 3 studies focused on autologous FMT, 12 clinical trials chose allogenic FMT, and 6 trials included both types for obesity treatment (Supplementary Table 8). The efficacy of autologous and allogeneic FMT differs considerably. Allogeneic FMT represents an “outside-in” microbial revolution aimed at comprehensively reconstructing a dysregulated microbiota using a healthy ecosystem. In contrast, autologous FMT functions as an “inside-out” microbiota maintenance strategy, where the core effect is stabilization rather than reconstruction, intended to protect and consolidate a healthy microbiota, thereby preventing regression to a pre-weight-loss state. Clinical trials have verified that allogenic FMT offers advantages in metabolic improvements, whereas autologous FMT demonstrates unique value in maintaining long-term weight management (Supplementary Table 8). Additionally, it has been reported that allogeneic FMT is predominantly associated with changes in amino acid concentrations, whereas autologous FMT appears to be linked to oxidative stress and lipid metabolites (Kootte et al., 2017). Kamer further investigated specific microbial changes associated with weight maintenance following autologous FMT, finding that low-abundance, non-core taxa can mediate successful weight loss and maintenance (Kamer et al., 2023). These non-core taxa are primarily beneficial bacteria involved in polyphenol metabolism and energy regulation, which contribute to the success of autologous FMT in sustaining weight loss and metabolic benefits.

Donor-recipient compatibility is a determinant variable in FMT. Ng SC (Ng et al., 2022) found that the participants with obesity required at least 20% microbial engraftment following allogenic FMT from lean donors. In contrast, autologous FMT involves almost identical strains in the recipient, making the introduced strains more compatible. Autologous FMT also offers the advantage of minimizing inter-individual pathogen transmission (Rinott et al., 2021a).

Intestinal microbiome changes appear more pronounced in the allogeneic group, although this long-term effect was weaker at 3 months. Yasaman Ghorbani revealed differences in intestinal microbiome changes at the genus level between the allogeneic and autologous groups (Ghorbani et al., 2023). In the early post-transplant phase, the allogeneic group showed a significant increase in *Bacteroides xylanisolvens* and *Lactococcus lactis*, along with a reduction in *Blautia obeum*, whereas only *Roseburia intestinalis* was significantly reduced at 3 months after FMT. In contrast, the autologous group exhibited a significant reduction in *Escherichia coli* during the early post-FMT phase. An increase in Bacteroidetes (e.g., *Alistipes putredinis*, *Bacteroides vulgatus*, and *Bacteroides uniformis*) was observed after autologous FMT treatment. Autologous FMT, particularly collected during the weight-loss phase, can maintain weight loss effect during the weight-regain phase. To further analyze the metabolic benefits following FMT, Zhang conducted a secondary analysis based on recipient response to FMT intervention. Responsiveness was characterized by more consistent engraftment of donor gut microbiota within the Firmicutes, such as *Faecalibacillus*, *Roseburia*, and Christensenellaceae (Zhang et al., 2024). Zuppi found that allogeneic FMT can even increase the abundance and diversity of gut phages (Zuppi et al., 2024).

### 4.6 Which are the targeted “specific” microbiomes in treating obesity?

Typical core species are defined as prevalent 66% of participants and capable of maintaining relative stability (Kamer et al., 2023). However, evidence suggests that there is no single “core” microbiome in healthy individuals (Kamer et al., 2023). The highly variable metabolic and microbial responses to FMT indicate a complex host-recipient dynamic relationship in population with obesity, especially in those with insulin resistance (Yu et al., 2020). Variables from donors and recipients can affect final clinical efficacy, raising the question of whether a targeted “specific” microbiome exists that could minimize variability and yield metabolic outcomes. Based on current research, individuals with obesity have been reported to exhibit reduced diversity of *Clostridium*, *Bifidobacterium*, *Coprococcus*, *A. muciniphila*, and *Streptococcus*, in contrast, alongside relative increases in *Bacteroides*, *Prevotella*, *Phascolarctobacterium*, and *Paraprevotella* (Quiroga et al., 2020). Among the various microbiomes associated with obesity, we sought to identify the most promising “specific” microbiomes that could contribute to successful weight loss and maintenance (Supplementary Table 8).

Gut microbiome composition matures by approximately 4 years of age and remains relatively stable throughout lifespan, encompassing primary phyla, such as Firmicutes, Bacteroidetes, Actinobacteria, and Proteobacteria (Nóbrega et al., 2025). Firmicutes and Bacteroidetes, constituting the dominant (90%) gut microbiota in healthy, and their relative abundance is closely associated with obesity. Individuals with obesity are characterized by a higher proportion of Firmicutes and a lower Bacteroidetes, resulting in an elevated Firmicutes/Bacteroidetes (F/B) ratio compared with the lean (Ley et al., 2006). While the F/B ratio was historically proposed as a signature of obesity-associated dysbiosis, mounting evidence has demonstrated that this ratio is highly variable, poorly reproducible, and insufficient to reflect functional or ecological status. Meanwhile, an increased *Prevotella*/*Bacteroides* (P/B) ratio has been reported in obesity (Dong et al., 2022). Ruan Y found significant engraftment of specific donor-derived taxa, such as *Phascolarctobacterium* and *Acidaminococcaceae* following FMT (Ruan et al., 2025). Notably, microbiome composition may predict metabolic responses (Zeevi et al., 2015). At genus level, *Prevotella* has been identified as the most effective strain for successful engraftment and metabolic improvement (Zhang et al., 2024). When P/B ratio exceeds 0.01, individuals consuming a high-fiber diet tend to show greater improvement in glucose metabolism (Hjorth et al., 2018). Birdshot also found that the adolescents with obesity exhibited more efficient gut implantation when donors were characterized by a high P/B ratio, suggesting that P/B ratio may serve as a potential “superimplanter” metric (Wilson et al., 2021). Successful microbial invasion and weight loss are more closely associated with Bacteroidetes, particularly *Alistipes putredinis* and *Bacteroides* (Rinott et al., 2021b). *Bacteroides* can proactively refine the intestinal environment, making it more hospitable for microbiota survival and engraftment. Its tolerance to hypoxia can enable *Bacteroides* to spread in a new gut environment during FMT (Wexler and Goodman, 2017). Therefore, *Bacteroides* represents one of the most promising targeted “specific” microbiomes.

*Akkermansia muciniphila*, the sole gram-negative anaerobic bacterium, resides in intestinal mucosa, particularly in the caecum, and accounts for 3%–5% of the gut microbiota in healthy individuals (Xu et al., 2020). *A. muciniphila* utilizes mucin as a source of nitrogen and carbon, subsequently decomposing oligosaccharides into acetic and propionic acids which can inhibit fat synthesis and stimulate glucagon-like peptide-1 (GLP-1) secretion (Xu et al., 2020; Shaheen et al., 2025). Furthermore, *A. muciniphila* activates G-protein-coupled receptors, promoting the differentiation of regulatory T cells (Tregs) and thereby suppressing excessive inflammatory responses via SCFAs (Plovier et al., 2017). By upregulating the expression of tight junction proteins and stimulating goblet cell proliferation and mucin secretion, this bacterium enhances gut barrier integrity and reduces lipopolysaccharide (LPS)-driven systemic inflammation (Hasani et al., 2021). As an important member of the Verrucomicrobia phylum, *A. muciniphila* can act synergistically with other phyla to exert anti-obesity effects through a multi-targeted network (Shaheen et al., 2025). Increased abundance of *A. muciniphila* is associated with significant compositional changes in the gut microbiome, particularly among its microbial allies (Shaheen et al., 2025). Compared with traditional probiotics (e.g., *Lactobacillus*, *Bifidobacterium*) derived from fermented foods, *A. muciniphila* is a native, core member of the human microbiota with a more direct and profound connection to host health (Shaheen et al., 2025). Numerous animal studies and preliminary human clinical trials have confirmed that supplementation with *A. muciniphila* is safe and well-tolerated. As a highly promising next-generation probiotic, its potential lies not only in the live bacterium but also in its specific effector proteins, such as the outer membrane protein Amuc_1100. This protein retains activity even after pasteurization and mimics many of the beneficial functions of live bacteria, paving the way for the development of more stable and safer “postbiotic” formulations (Plovier et al., 2017). Precise strain selection is crucial for future applications but should be grounded in functional and ecological metrics.

## 5 Discussion

Fecal microbiota transplantation has emerged as a promising therapeutic strategy in obesity. This study conducted a systematic literature search on FMT in obesity to map the current research landscape and identify potential hotspots in this field.

### 5.1 Functional insights into host-microbe interactions in FMT for obesity

The therapeutic value of FMT arises primarily from functional recovery of disrupted microbial pathways to improve insulin sensitivity, reduce systemic inflammation, normalize energy harvesting, and restore homeostatic host–microbe communication. Key functional dimensions underlying FMT efficacy in obesity are summarized below.

#### 5.1.1 SCFAs: beyond energy substrates

Short-chain fatty acids, primarily acetate, propionate, and butyrate, are produced via anaerobic fermentation of dietary fibers by gut commensals. They are not merely energy sources for colonocytes but act as signaling molecules regulating host metabolism (Liu, 2025). Butyrate, for instance, serves as the primary fuel for intestinal epithelial cells and enhances gut barrier integrity by upregulating tight junction proteins (e.g., claudin-1, occludin) (Luo et al., 2022; Yang et al., 2025). Propionate and acetate are absorbed into the portal circulation and reach the liver, where they modulate gluconeogenesis and lipogenesis (Luo et al., 2022). More importantly, SCFAs activate G-protein-coupled receptors (GPCRs), particularly FFAR2 (GPR43) and FFAR3 (GPR41), expressed on enteroendocrine L-cells, adipocytes, and immune cells (Jiao et al., 2020; Liu, 2025). Activation of FFAR3 stimulates the secretion of GLP-1 and peptide YY (PYY), leading to enhanced insulin secretion, reduced appetite, and delayed gastric emptying (Jiao et al., 2020; Vishwakarma et al., 2025; Liu, 2025). In individuals with obesity, reduced SCFA-producing bacteria (e.g., *Faecalibacterium prausnitzii*, *Roseburia intestinalis*, *Eubacterium rectale*) have been consistently observed (Cui et al., 2025; Vishwakarma et al., 2025). FMT from lean donors has been shown to restore SCFA levels, although the magnitude and duration of restoration vary considerably across studies (Cui et al., 2025).

#### 5.1.2 BA metabolism and signaling

Bile acids are synthesized in the liver from cholesterol and undergo secondary modification by gut microbiota, including deconjugation, dehydroxylation, and epimerization (Chen and Gong, 2025). The gut microbiota regulates the BA pool composition, which in turn modulates host metabolism via the farnesoid X receptor (FXR) and Takeda G-protein-coupled receptor 5 (TGR5) (Chen and Gong, 2025; Cui et al., 2025; Liu, 2025). Activation of TGR5 in enteroendocrine L-cells promotes GLP-1 secretion, while FXR signaling in the liver and ileum influences triglyceride and glucose homeostasis. Dysbiosis in obesity is associated with altered BA profiles, such as an increased ratio of primary to secondary BAs. FMT from lean donors has been reported to partially normalize BA composition, although the precise microbial taxa responsible for 7α-dehydroxylation (e.g., *Clostridium scindens*, *Bacteroides* species) remain incompletely characterized (Liu, 2025).

#### 5.1.3 Trimethylamine N-Oxide (TMAO) pathway

Choline and L-carnitine from dietary animal products are converted by gut microbiota to trimethylamine (TMA), which is then absorbed and oxidized by hepatic flavin-containing monooxygenases (FMOs) to TMAO (Stoian et al., 2026). Elevated TMAO levels are associated with insulin resistance, adipose tissue inflammation, and increased cardiovascular risk. Notably, certain bacterial taxa enriched in individuals with obesity (e.g., *Prevotella copri*, Firmicutes) exhibit higher TMA-producing capacity (Smits et al., 2018). Whether FMT from lean donors consistently reduces TMAO production remains unclear, as this pathway is highly diet-sensitive and donor-specific.

#### 5.1.4 Endotoxemia and metabolic inflammation

Obesity-related dysbiosis often leads to increased intestinal permeability, allowing translocation of bacterial LPS into the systemic circulation–a state termed “metabolic endotoxemia.” LPS triggers Toll-like receptor 4 (TLR4) signaling on macrophages and adipocytes, promoting pro-inflammatory cytokine release (e.g., TNF-α, IL-6, IL-1β) and insulin resistance (Cui et al., 2025). Several FMT trials have observed reductions in circulating LPS-binding protein (LBP) and inflammatory markers, particularly when donors were rich in *A. muciniphila* and *Bifidobacterium* species, both known to reinforce mucus layer integrity and compete with Proteobacteria (Liu, 2025).

#### 5.1.5 Host-microbe signaling through the gut-brain axis

The gut microbiota communicates with the central nervous system via neural, endocrine, and immune pathways (Castells-Nobau et al., 2026). Gut microbes produce or modulate neurotransmitters (e.g., GABA, serotonin, dopamine) and precursors (e.g., tryptophan) (Hartstra et al., 2020). For example, *Lactobacillus* and *Bifidobacterium* species can convert dietary glutamate to GABA, while *Candida*, *Escherichia*, and *Enterococcus* produce serotonin from tryptophan. These molecules influence appetite regulation, satiety, and rewards processing. FMT-induced changes in microbial tryptophan metabolism may affect kynurenine pathway activity, which is linked to low-grade inflammation and mood disorders commonly comorbid with obesity (Hartstra et al., 2020). However, direct evidence linking FMT to gut-brain axis modulation remains sparse.

### 5.2 Increasing focus on FMT and geographical underrepresentation calls for more international cooperation

Between 2006 and 2012, the field underwent a pivotal transformation from descriptive association studies to mechanistic and functional investigations. Early work established the correlative link between gut microbiota and obesity (Ley et al., 2006; Turnbaugh et al., 2006), while subsequent gnotobiotic models provided causal evidence linking microbial composition to metabolic phenotypes (Qin et al., 2010, 2012; Vrieze et al., 2012; Ridaura et al., 2013). The elucidation of mechanisms–particularly the role of microbial metabolites, metabolic endotoxemia via the LPS-TLR4 pathway, and modulation of enteroendocrine hormones (e.g., GLP-1, PYY)–marked the transition from descriptive “who is there” studies to mechanistic “what do they do” investigations (Cani et al., 2008). These breakthroughs established the functional framework for microbiota mediated obesity pathogenesis. During this same period, the first human proof-of-concept trial demonstrated that FMT from lean donors improved insulin sensitivity in individuals with metabolic syndrome (Vrieze et al., 2012), thereby laying the groundwork for subsequent clinical translation.

Although China contributed the largest number of publications (47.6%), institutional collaborations were domestically concentrated with limited cross border partnerships compared with European and North American groups. This pattern may reflect regional research priorities, funding mechanisms, and a focus on early-stage clinical translation, with fewer multinational consortia. First, language and cultural barriers, combined with stringent regulations on cross-border fecal sample transport, hinder multi-center international trials and sample-sharing. Second, China’s distinctive dietary patterns and high antibiotic exposure mean that gut microbial structures in Chinese populations differ considerably from those in Western cohorts, justifying a localized precision-medicine approach. Consequently, while China leads in publication volume, its share of high-impact studies defining mechanistic concepts or shaping international guidelines remains relatively modest, highlighting an opportunity for strategic global engagement. In contrast, the USA, Netherlands, UK, and other European countries formed extensive collaborative networks (Figure 2B), facilitating shared clinical resources, standardized protocols, and multi-center validation. Strengthening international cooperation will be critical to improving sample sizes, standardizing FMT procedures, and enhancing the generalizability of findings across ethnic and geographic populations. The near absence of publications from Southeast Asia and South America is a major knowledge gap. In Southeast Asia, obesity rates are rising rapidly–overweight prevalence among young adults reaches up to 28.8% in some developing countries in the region–yet gut microbiome research remains nascent, hampered by limited genomic infrastructure, competing infectious disease priorities, and small sample sizes (Somnuk et al., 2023). Pioneering descriptive studies from Thailand have begun to characterize local microbiota signatures, but no FMT trials for obesity have been conducted in this region (Jinatham et al., 2018). In South America, despite the region’s exceptional ethnic admixture, dietary diversity, and dual burden of infectious and non-communicable diseases, microbiome research has been severely limited owing to insufficient funding, imported reagent costs, and economic instability (Reyes et al., 2025). Initiatives such as the South American MicroBiome Archive (saMBA), a landmark project that compiled one of the largest South American gut metagenomic datasets, and the Latinbiota Consortium, which aims to build local research capacity and ensure equitable participation in global microbiome science, represent critically needed steps (Reyes et al., 2025; Valderrama et al., 2025). This geographical underrepresentation is not merely an issue of scientific equity; it poses a substantive risk that FMT protocols and therapeutic products optimized for European/North American populations may fail or even produce adverse outcomes when applied to individuals with distinct genetic, dietary, and lifestyle backgrounds. Additionally, variable obesity epidemiology, dietary patterns, and genetic backgrounds in these regions may further delay research initiation (Valderrama et al., 2025). Therefore, strategic investment in capacity-building, international consortium funding, and open-access metadata standardization for underrepresented regions is urgently needed to ensure the global generalizability of FMT-based therapies.

### 5.3 High-quality clinical trials are needed to establish standards for clinical application

Although FMT shows huge potential in the treatment of obesity, numerous variables remain to be investigated before widespread clinical application (Vrieze et al., 2012; Ridaura et al., 2013; Kootte et al., 2017; Allegretti et al., 2020; Yu et al., 2020; Leong et al., 2020; Mocanu et al., 2021). The absence of standardized protocols indicates that the therapeutic claims regarding FMT in obesity should be interpreted with caution due to the transient nature of engraftment and the high inter-individual variability in metabolic responses. Several key aspects warrant further investigation.

First, donor selection criteria vary widely across trials, ranging from rigorously screened lean donors to unspecified or autologous donors. Donor microbiome composition differs substantially between studies, yet no standardized donor-recipient matching criteria exist. Recipient baseline characteristics are inconsistently reported, making it difficult to identify predictors of response. Second, confounding variables such as concomitant diet, physical activity, antibiotic use, and bowel preparation protocols were inconsistently reported across studies, making it difficult to isolate the true effect of FMT. Third, the lack of standardized outcome measures hinders cross-study comparisons and meta-analyses. The time points for outcome assessment also vary, and most trials lack long-term follow-up. Similarly, whether changes in stool microbiota faithfully reflect alterations in microbiota across different gut regions is yet to be determined. Zhang (Zhang et al., 2022) found that FMT led to an increased abundance of *Bacteroides* in the colonic mucosal microbiome in the successful weight-loss individuals with obesity, whereas the duodenal mucosal microbiome remained stable. Therefore, when reporting intestinal microbiome changes, the researchers should report detailed information regarding the specific gut regions studied. Finally, the majority of clinical trials to date are small, open-label, and lack sham-controlled (placebo) arms. Only a handful of randomized, double-blind, placebo-controlled trials have been conducted. Small sample sizes increase the risk of type II errors and limit subgroup analyses to identify responders versus non-responders.

Given these uncertainties, the current evidence does not support the routine use of FMT for obesity outside of well-controlled clinical trials. When assessing the clinical efficacy of FMT, study designs should account for background diet and host characteristics and report more evaluation index. The optimal dosage, administration frequency, long-term impacts and potential adverse reactions remain to be elucidated (Vrieze et al., 2012; Kootte et al., 2017). High-quality, large-scale clinical trials are urgently needed to establish clinical application guidelines and minimize the risk of pathogen transmission.

### 5.4 Potential trend: from total fecal transplantation to a targeted precision strategy

The evolution of keyword clusters from 2008 to 2025 reveals a clear trajectory: early clusters (2008–2012) focused on “diet-induced obesity” and “gut microbiota composition”; intermediate clusters (2013–2018) emphasized “inflammation,” “insulin resistance,” and “short-chain fatty acids”; and recent clusters (2019–2025) have shifted toward “targeting gut microbiota,” “oral supplementation,” and “precision microbiome.” This progression reflects a maturation from basic taxonomic description to mechanism-based interventions and, finally, to clinical translation. These temporal changes align with the maturation of microbiome science from observational association to mechanism driven precision therapy.

#### 5.4.1 Evidence-based strategies for precision FMT in obesity

To move beyond crude fecal transplantation toward precision microbiome medicine, we propose a three-component strategy that integrates defined microbial consortia, donor–recipient matching criteria, and functional targeting based on baseline host–microbiome profiling.

Defined microbial consortia. Instead of untargeted whole-stool FMT, we propose a modular, defined consortium approach.

A core consortium should include:

- *Bacteroides uniformis* – facilitates engraftment by tolerating hypoxia and restructuring recipient gut environment (Wexler and Goodman, 2017; Rinott et al., 2021b).
- *Akkermansia muciniphila* – restores mucus layer, reduces endotoxemia, and improves insulin sensitivity via Amuc_1100 (Everard et al., 2013; Depommier et al., 2019).
- *Faecalibacterium prausnitzii* – a major butyrate producer with anti-inflammatory properties (Miquel et al., 2013).

Optional functional modules can be added based on recipient deficits:

- SCFA-boosting module: *Roseburia intestinalis* + *Eubacterium rectale* (Cui et al., 2025; Vishwakarma et al., 2025).
- BA-modulating module: *Clostridium scindens* (Chen and Gong, 2025; Liu, 2025).
- Anti-inflammatory module: *Bifidobacterium longum* + *Lactobacillus plantarum* (Cui et al., 2025; Liu, 2025).

Clinical trials have begun testing such consortia. We recommend that future studies adopt tiered consortium design where the core is fixed and modules are individualized.

Donor-recipient matching criteria. Clinical outcomes strongly depend on donor compatibility rather than donor leanness alone. Developing a personalized donor-recipient matching system based on microbial community profiles represents a shift from “total fecal transplantation” toward defined and personalized microbial consortia. Based on existing evidence, we propose a forward-looking, evidence-informed blueprint for quantifiable matching metrics.

- Baseline recipient microbial diversity: Recipients with low Shannon diversity (<3.0) or low gene richness (<500,000 genes) show greater engraftment success (Vrieze et al., 2012; Kootte et al., 2017).
- P/B ratio: A donor P/B ratio > 0.01 is associated with improved glucose metabolism in high-fiber diet contexts (Hjorth et al., 2018; Wilson et al., 2021). We suggest donor P/B ratio as a “superimplanter” metric.
- Donor *A. muciniphila* abundance: Donors with >1% *A. muciniphila* are preferred, as this bacterium is often depleted in obesity (Everard et al., 2013; Depommier et al., 2019).
- β−diversity compatibility: Weighted UniFrac distance between donor and recipient should ideally be <0.3, reflecting moderate dissimilarity without excessive mismatch (Wilson et al., 2021).
- Functional gene complementarity: Donor and recipient should be matched for metabolic pathways. For example, if the recipient has low butyrate synthesis potential, the donor should be enriched in butyrate kinase genes (*buk*, *ptb*) (Li et al., 2016; Podlesny et al., 2022).

A scoring system can be developed using these metrics to rank candidate donors. While not yet validated in prospective trials, such a system represents a clear departure from random donor selection.

Functional targeting based on host phenotyping. Instead of broad taxonomic modification, precision FMT should target specific disrupted pathways: SCFA production to enhance GPR43/41 signaling and appetite suppression (Jiao et al., 2020; Liu, 2025); BA metabolism to activate FXR–TGR5-dependent thermogenesis and lipid control (Chen and Gong, 2025); Gut barrier restoration to reduce LPS-driven metabolic inflammation (Cui et al., 2025); Gut-brain axis modulation to normalize food rewards and energy expenditure (Hartstra et al., 2020).

Contemporary research prioritizes functional and ecological metrics such as pathway activity, metabolite profiles, engraftment stability, and ecosystem resilience, which better reflect biological mechanisms and clinical responses (Liu, 2025).

#### 5.4.2 Ethics, safety, and accessibility of oral FMT capsules

As a “living biological drug,” FMT is still under development, and the psychological acceptability of using feces as a “medication” remains a major obstacle, making the optimization of delivery method imperative. The emergence of “#13 oral supplementation” (Figure 4C and Table 4) indicates a future trend. Oral FMT capsules address key barriers to clinical adoption. Ethically, cFMT improves acceptability by avoiding invasive endoscopic procedures and reduces psychological stigma associated with fecal based therapies. Clinically, multiple trials confirm that cFMT has noninferior efficacy to endoscopic delivery with favorable safety profiles, mostly mild and self-limiting adverse events. From an accessibility perspective, cFMT enables repeated administration, simplifies logistics, reduces costs, and supports scalable, pharmaceutical grade production. However, regulatory frameworks for cFMT as a live biotherapeutic product remain under development, and long-term safety data are still lacking. Standardized manufacturing, long term safety monitoring, and donor screening regulations are still required to ensure widespread clinical application.

#### 5.4.3 Contradiction between uncontrolled diet and proposed diet FMT combination

Current studies have attempted to identify internal and external factors that influence microbiome composition and diversity (Zhernakova et al., 2016; McBurney and Cho, 2024), revealing that dietary factors and environmental factors may outweigh genetic factors. Consequently, clusters such as “#0 food preference alteration,” “#1 Tibetan tea,” “#14 gut microbiota transplantation quercetin” (Figure 4C and Table 4), and “#3 resveratrol” (Figure 2C) may reflect a preference for combining dietary arrangement in weight management.

Most clinical trials do not standardize dietary intake, yet preclinical and translational evidence strongly supports that dietary composition profoundly modulates FMT engraftment and efficacy. Current evidence remains inconclusive owing to heterogeneous dietary protocols. High-fiber, low-fat diets provide preferred substrates for beneficial microbes, enhance colonization stability, and sustain metabolic improvements. Conversely, high-fat, low-fiber diets impede engraftment and promote dysbiosis recurrence. Future trials should standardize dietary counseling or provide controlled meals during the peri-transplantation period to isolate the FMT effect. Until such data emerge, the combination of FMT with unstructured lifestyle advice cannot be recommended over FMT alone. Future trials must incorporate standardized lifestyle interventions to improve efficacy consistency and translate FMT into robust clinical outcomes.

### 5.5 Strengths and limitations

This study presents the first bibliometric analysis of FMT in the treatment of obesity, conducted using VOSviewer and CiteSpace software, with the aim of providing a systematic, quantitative, and objective description and analysis. We further conducted a mini-review of clinical progress to examine the practical status concerning several key issues that require validation through high-quality trials. Nevertheless, several limitations should be acknowledged. First, due to data harmonization in metadata formats, citation indexing and author name disambiguation, we did not directly incorporate multiple databases (WoSCC and PubMed), which may not fully capture all relevant publications. Second, only English-language papers were included, potentially introducing linguistic bias, and excluding other high-quality studies. Third, the document types were restricted to articles and reviews, which may have resulted in incomplete data collection from other publication formats. Fourth, although we performed rigorous keyword normalization, residual keyword variability may persist due to differences in indexing practices between WoSCC and PubMed, as well as author-driven keyword selection bias. This may slightly affect co-occurrence frequencies and burst detection. Therefore, our keyword-based findings should be interpreted as indicative of general trends rather than precise quantitative measures. Finally, delays in the online publication of records may have introduced inaccuracies in citation and collaboration analyses. Future studies should consider including non-English manuscripts and a broader range of document types to provide more comprehensive and up-to-date perspectives.

## 6 Conclusion

Fecal microbiota transplantation is a promising but still experimental strategy for obesity management, although current evidence shows that clinical benefits are often transient and highly variable. Targeted oral microbiota supplementation may emerge as a future direction. More high-quality, large-scale clinical trials are urgently needed to establish standardized outcome measures, define optimal donor-recipient matching criteria, and systematically evaluate long-term safety and efficacy before FMT can be considered for routine clinical application.

## Data Availability

The original contributions presented in this study are included in the article/Supplementary material, further inquiries can be directed to the corresponding authors.

## References

[B1] Alang N. Kelly C. R. (2015). Weight gain after fecal microbiota transplantation. *Open Forum Infect. Dis.* 2:ofv004. 10.1093/ofid/ofv004 26034755 PMC4438885

[B2] Allegretti J. R. Kassam Z. Mullish B. H. Chiang A. Carrellas M. Hurtado J. et al. (2020). Effects of fecal microbiota transplantation with oral capsules in obese patients. *Clin. Gastroenterol. Hepatol.* 18 855–863.e2. 10.1016/j.cgh.2019.07.006 31301451

[B3] Allen J. M. Mailing L. J. Niemiro G. M. Moore R. Cook M. D. White B. A. et al. (2018). Exercise alters gut microbiota composition and function in lean and obese humans. *Med. Sci. Sports Exerc.* 50 747–757. 10.1249/MSS.0000000000001495 29166320

[B4] Anhê F. F. Roy D. Pilon G. Dudonné S. Matamoros S. Varin T. V. et al. (2015). A polyphenol-rich cranberry extract protects from diet-induced obesity, insulin resistance and intestinal inflammation in association with increased *Akkermansia spp*. population in the gut microbiota of mice. *Gut* 64 872–883. 10.1136/gutjnl-2014-307142 25080446

[B5] Apovian C. M. (2016). Obesity: Definition, comorbidities, causes, and burden. *Am. J. Manag. Care* 22 s176–s185. 27356115

[B6] Bäckhed F. Ding H. Wang T. Hooper L. V. Koh G. Y. Nagy A. et al. (2004). The gut microbiota as an environmental factor that regulates fat storage. *Proc. Natl. Acad. Sci. U. S. A.* 101 15718–15723. 10.1073/pnas.0407076101 15505215 PMC524219

[B7] Beam A. Clinger E. Hao L. (2021). Effect of diet and dietary components on the composition of the gut microbiota. *Nutrients* 13:2795. 10.3390/nu13082795 34444955 PMC8398149

[B8] Boulangé C. L. Neves A. L. Chilloux J. Nicholson J. K. Dumas M.-E. (2016). Impact of the gut microbiota on inflammation, obesity, and metabolic disease. *Genome Med.* 8:42. 10.1186/s13073-016-0303-2 27098727 PMC4839080

[B9] Campbell S. C. Wisniewski P. J. Noji M. McGuinness L. R. Häggblom M. M. Lightfoot S. A. et al. (2016). The effect of diet and exercise on intestinal integrity and microbial diversity in mice. *PLoS One* 11:e0150502. 10.1371/journal.pone.0150502 26954359 PMC4783017

[B10] Cani P. D. Amar J. Iglesias M. A. Poggi M. Knauf C. Bastelica D. et al. (2007). Metabolic endotoxemia initiates obesity and insulin resistance. *Diabetes* 56 1761–1772. 10.2337/db06-1491 17456850

[B11] Cani P. D. Bibiloni R. Knauf C. Waget A. Neyrinck A. M. Delzenne N. M. et al. (2008). Changes in gut microbiota control metabolic endotoxemia-induced inflammation in high-fat diet-induced obesity and diabetes in mice. *Diabetes* 57 1470–1481. 10.2337/db07-1403 18305141

[B12] Carbajo-Pescador S. Porras D. García-Mediavilla M. V. Martínez-Flórez S. Juarez-Fernández M. Cuevas M. J. et al. (2019). Beneficial effects of exercise on gut microbiota functionality and barrier integrity, and gut-liver crosstalk in an in vivo model of early obesity and non-alcoholic fatty liver disease. *Dis. Model Mech.* 12:dmm039206. 10.1242/dmm.039206 30971408 PMC6550047

[B13] Carlucci C. Petrof E. O. Allen-Vercoe E. (2016). Fecal microbiota-based therapeutics for recurrent clostridium difficile infection, ulcerative colitis and obesity. *eBioMedicine* 13 37–45. 10.1016/j.ebiom.2016.09.029 27720396 PMC5264253

[B14] Castells-Nobau A. Fumagalli A. Del Castillo-Izquierdo Á Rosell-Díaz M. de la Vega-Correa L. Samulënaitë S. et al. (2026). Gut microbial modulation of 3-hydroxyanthranilic acid and dopaminergic signalling influences attention in obesity. *Gut* 75 705–724. 10.1136/gutjnl-2025-336391 41015495 PMC13018844

[B15] Chen C. (2004). Searching for intellectual turning points: Progressive knowledge domain visualization. *Proc. Natl. Acad. Sci. U. S. A.* 101 (Suppl. 1), 5303–5310. 10.1073/pnas.0307513100 14724295 PMC387312

[B16] Chen D. B. Gao H. Lü L. Zhou T. (2013). Identifying influential nodes in large-scale directed networks: The role of clustering. *PLoS One* 8:e77455. 10.1371/journal.pone.0077455 24204833 PMC3814409

[B17] Chen F. Gong L. (2025). Bile acid-microbiota interactions in cardiometabolic diseases: Mechanisms and emerging therapeutic approaches. *Front. Microbiol.* 16:1689026. 10.3389/fmicb.2025.1689026 41480102 PMC12753514

[B18] Cui X. Yuan Q. Long J. Zhou J. (2025). Recent advances in gut microbiota-mediated regulation of fat deposition and metabolic disorders. *Microb. Res. Rep.* 4:31. 10.20517/mrr.2025.25 41133095 PMC12540052

[B19] David L. A. Maurice C. F. Carmody R. N. Gootenberg D. B. Button J. E. Wolfe B. E. et al. (2014). Diet rapidly and reproducibly alters the human gut microbiome. *Nature* 505 559–563. 10.1038/nature12820 24336217 PMC3957428

[B20] De Vadder F. Kovatcheva-Datchary P. Goncalves D. Vinera J. Zitoun C. Duchampt A. et al. (2014). Microbiota-generated metabolites promote metabolic benefits via gut-brain neural circuits. *Cell* 156 84–96. 10.1016/j.cell.2013.12.016 24412651

[B21] Depommier C. Everard A. Druart C. Plovier H. Van Hul M. Vieira-Silva S. et al. (2019). Supplementation with akkermansia muciniphila in overweight and obese human volunteers: A proof-of-concept exploratory study. *Nat. Med.* 25 1096–1103. 10.1038/s41591-019-0495-2 31263284 PMC6699990

[B22] Ding X. Yang Z. (2022). Knowledge mapping of platform research: A visual analysis using VOSviewer and CiteSpace. *Electron. Commer. Res.* 22 787–809. 10.1007/s10660-020-09410-7

[B23] Dong T. S. Guan M. Mayer E. A. Stains J. Liu C. Vora P. et al. (2022). Obesity is associated with a distinct brain-gut microbiome signature that connects prevotella and bacteroides to the brain’s reward center. *Gut Microbes* 14:2051999. 10.1080/19490976.2022.2051999 35311453 PMC8942409

[B24] Endalifer M. L. Diress G. (2020). Epidemiology, predisposing factors, biomarkers, and prevention mechanism of obesity: A systematic review. *J. Obes.* 2020:6134362. 10.1155/2020/6134362 32566274 PMC7281819

[B25] Everard A. Belzer C. Geurts L. Ouwerkerk J. P. Druart C. Bindels L. B. et al. (2013). Cross-talk between akkermansia muciniphila and intestinal epithelium controls diet-induced obesity. *Proc. Natl. Acad. Sci. U. S. A.* 110 9066–9071. 10.1073/pnas.1219451110 23671105 PMC3670398

[B26] Fan Y. Pedersen O. (2021). Gut microbiota in human metabolic health and disease. *Na. Rev. Microbiol.* 19 55–71. 10.1038/s41579-020-0433-9 32887946

[B27] Fukuyama J. Rumker L. Sankaran K. Jeganathan P. Dethlefsen L. Relman D. A. et al. (2017). Multidomain analyses of a longitudinal human microbiome intestinal cleanout perturbation experiment. *PLoS Comput. Biol.* 13:e1005706. 10.1371/journal.pcbi.1005706 28821012 PMC5576755

[B28] Ghorbani Y. Schwenger K. J. P. Sharma D. Jung H. Yadav J. Xu W. et al. (2023). Effect of faecal microbial transplant via colonoscopy in patients with severe obesity and insulin resistance: A randomized double-blind, placebo-controlled phase 2 trial. *Diab. Obes. Metab.* 25 479–490. 10.1111/dom.14891 36239189

[B29] Goodrich J. K. Waters J. L. Poole A. C. Sutter J. L. Koren O. Blekhman R. et al. (2014). Human genetics shape the gut microbiome. *Cell* 159 789–799. 10.1016/j.cell.2014.09.053 25417156 PMC4255478

[B30] Gough E. Shaikh H. Manges A. R. (2011). Systematic review of intestinal microbiota transplantation (fecal bacteriotherapy) for recurrent clostridium difficile infection. *Clin. Infect. Dis.* 53 994–1002. 10.1093/cid/cir632 22002980

[B31] Hartstra A. V. Schüppel V. Imangaliyev S. Schrantee A. Prodan A. Collard D. et al. (2020). Infusion of donor feces affects the gut-brain axis in humans with metabolic syndrome. *Mol. Metab.* 42:101076. 10.1016/j.molmet.2020.101076 32916306 PMC7536740

[B32] Hasani A. Ebrahimzadeh S. Hemmati F. Khabbaz A. Hasani A. Gholizadeh P. (2021). The role of akkermansia muciniphila in obesity, diabetes and atherosclerosis. *J. Med. Microbiol.* 70. 10.1099/jmm.0.001435 34623232

[B33] Hjorth M. F. Roager H. M. Larsen T. M. Poulsen S. K. Licht T. R. Bahl M. I. et al. (2018). Pre-treatment microbial prevotella-to-bacteroides ratio, determines body fat loss success during a 6-month randomized controlled diet intervention. *Int. J. Obes.* 42 580–583. 10.1038/ijo.2017.220 28883543 PMC5880576

[B34] Jiao A. Yu B. He J. Yu J. Zheng P. Luo Y. et al. (2020). Short chain fatty acids could prevent fat deposition in pigs via regulating related hormones and genes. *Food Funct.* 11 1845–1855. 10.1039/c9fo02585e 32067021

[B35] Jinatham V. Kullawong N. Kespechara K. Gentekaki E. Popluechai S. (2018). Comparison of gut microbiota between lean and obese adult thai individuals. *Microbiol. Biotechnol. Lett.* 46 277–287. 10.4014/mbl.1711.11003

[B36] Kamer O. Rinott E. Tsaban G. Kaplan A. Yaskolka Meir A. Zelicha H. et al. (2023). Successful weight regain attenuation by autologous fecal microbiota transplantation is associated with non-core gut microbiota changes during weight loss; randomized controlled trial. *Gut Microbes* 15:2264457. 10.1080/19490976.2023.2264457 37796016 PMC10557561

[B37] Kao D. Roach B. Silva M. Beck P. Rioux K. Kaplan G. G. et al. (2017). Effect of oral capsule– vs colonoscopy-delivered fecal microbiota transplantation on recurrent clostridium difficile infection. *JAMA* 318 1985–1993. 10.1001/jama.2017.17077 29183074 PMC5820695

[B38] Karlsson F. H. Tremaroli V. Nookaew I. Bergström G. Behre C. J. Fagerberg B. et al. (2013). Gut metagenome in european women with normal, impaired and diabetic glucose control. *Nature* 498 99–103. 10.1038/nature12198 23719380

[B39] Kelley G. A. Kelley K. S. Pate R. R. (2014). Effects of exercise on BMI z-score in overweight and obese children and adolescents: A systematic review with meta-analysis. *BMC Pediatr.* 14:225. 10.1186/1471-2431-14-225 25204857 PMC4180550

[B40] König J. Siebenhaar A. Högenauer C. Arkkila P. Nieuwdorp M. Norén T. et al. (2017). Consensus report: Faecal microbiota transfer – clinical applications and procedures. *Aliment Pharmacol. Ther.* 45 222–239. 10.1111/apt.13868 27891639 PMC6680358

[B41] Kootte R. S. Levin E. Salojärvi J. Smits L. P. Hartstra A. V. Udayappan S. D. et al. (2017). Improvement of insulin sensitivity after lean donor feces in metabolic syndrome is driven by baseline intestinal microbiota composition. *Cell Metab.* 26 611–619.e6. 10.1016/j.cmet.2017.09.008 28978426

[B42] Lahtinen P. Juuti A. Luostarinen M. Niskanen L. Liukkonen T. Tillonen J. et al. (2022). Effectiveness of fecal microbiota transplantation for weight loss in patients with obesity undergoing bariatric surgery: A randomized clinical trial. *JAMA Netw. Open* 5:e2247226. 10.1001/jamanetworkopen.2022.47226 36525272 PMC9856235

[B43] Le Chatelier E. Nielsen T. Qin J. Prifti E. Hildebrand F. Falony G. et al. (2013). Richness of human gut microbiome correlates with metabolic markers. *Nature* 500 541–546. 10.1038/nature12506 23985870

[B44] Leong K. S. W. Jayasinghe T. N. Wilson B. C. Derraik J. G. B. Albert B. B. Chiavaroli V. et al. (2020). Effects of fecal microbiome transfer in adolescents with obesity: The gut bugs randomized controlled trial. *JAMA Net. Open* 3:e2030415. 10.1001/jamanetworkopen.2020.30415 33346848 PMC7753902

[B45] Ley R. E. Bäckhed F. Turnbaugh P. Lozupone C. A. Knight R. D. Gordon J. I. (2005). Obesity alters gut microbial ecology. *Proc. Natl. Acad. Sci. U. S. A.* 102 11070–11075. 10.1073/pnas.0504978102 16033867 PMC1176910

[B46] Ley R. E. Turnbaugh P. J. Klein S. Gordon J. I. (2006). Microbial ecology: Human gut microbes associated with obesity. *Nature* 444 1022–1023. 10.1038/4441022a 17183309

[B47] Li S. S. Zhu A. Benes V. Costea P. I. Hercog R. Hildebrand F. et al. (2016). Durable coexistence of donor and recipient strains after fecal microbiota transplantation. *Science* 352 586–589. 10.1126/science.aad8852 27126044

[B48] Liou A. P. Paziuk M. Luevano J. M. Jr. Machineni S. Turnbaugh P. J. Kaplan L. M. (2013). Conserved shifts in the gut microbiota due to gastric bypass reduce host weight and adiposity. *Sci. Transl. Med.* 5:178ra41. 10.1126/scitranslmed.3005687 23536013 PMC3652229

[B49] Liu S. (2025). Mechanisms of gut microbiota in host fat deposition: Metabolites, signaling pathways, and translational applications. *Front. Microbiol.* 16:1675155. 10.3389/fmicb.2025.1675155 41488302 PMC12756826

[B50] Luo P. Lednovich K. Xu K. Nnyamah C. Layden B. T. Xu P. (2022). Central and peripheral regulations mediated by short-chain fatty acids on energy homeostasis. *Transl. Res.* 248 128–150. 10.1016/j.trsl.2022.06.003 35688319 PMC12553404

[B51] McBurney M. I. Cho C. E. (2024). Understanding the role of the human gut microbiome in overweight and obesity. *Ann. N. Y. Acad. Sci.* 1540 61–88. 10.1111/nyas.15215 39283061

[B52] Miquel S. Martín R. Rossi O. Bermúdez-Humarán L. G. Chatel J. M. Sokol H. et al. (2013). *Faecalibacterium prausnitzii* and human intestinal health. *Curr. Opin. Microbiol.* 16 255–261. 10.1016/j.mib.2013.06.003 23831042

[B53] Mirakyan M. (2021). ABCDE: Approximating betweenness-centrality ranking with progressive-DropEdge. *PeerJ. Comp. Sci.* 7:e699. 10.7717/peerj-cs.699 34604524 PMC8444073

[B54] Mocanu V. Zhang Z. Deehan E. C. Kao D. H. Hotte N. Karmali S. et al. (2021). Fecal microbial transplantation and fiber supplementation in patients with severe obesity and metabolic syndrome: A randomized double-blind, placebo-controlled phase 2 trial. *Nat. Med.* 27 1272–1279. 10.1038/s41591-021-01399-2 34226737

[B55] Mohajeri M. H. Brummer R. J. M. Rastall R. A. Weersma R. K. Harmsen H. J. M. Faas M. et al. (2018). The role of the microbiome for human health: From basic science to clinical applications. *Eur. J. Nutr.* 57 1–14. 10.1007/s00394-018-1703-4 29748817 PMC5962619

[B56] Ncd Risk Factor Collaboration (Ncd-RisC) (2024). Worldwide trends in underweight and obesity from 1990 to 2022: A pooled analysis of 3663 population-representative studies with 222 million children, adolescents, and adults. *Lancet* 403 1027–1050. 10.1016/S0140-6736(23)02750-2 38432237 PMC7615769

[B57] Neves A. L. Chilloux J. Sarafian M. H. Rahim M. B. Boulangé C. L. Dumas M. E. (2015). The microbiome and its pharmacological targets: Therapeutic avenues in cardiometabolic diseases. *Curr. Opin. Pharmacol.* 25 36–44. 10.1016/j.coph.2015.09.013 26531326

[B58] Ng S. C. Xu Z. Mak J. W. Y. Yang K. Liu Q. Zuo T. et al. (2022). Microbiota engraftment after faecal microbiota transplantation in obese subjects with type 2 diabetes: A 24-week, double-blind, randomised controlled trial. *Gut* 71 716–723. 10.1136/gutjnl-2020-323617 33785557

[B59] Nicholson J. K. Wilson I. D. (2003). Opinion: Understanding “global” systems biology: Metabonomics and the continuum of metabolism. *Nat. Rev. Drug Discovery* 2 668–676. 10.1038/nrd1157 12904817

[B60] Nicholson J. K. Holmes E. Wilson I. D. (2005). Gut microorganisms, mammalian metabolism and personalized health care. *Nat. Rev. Microbiol.* 3 431–438. 10.1038/nrmicro1152 15821725

[B61] Nóbrega R. Costa C. F. F. A. Cerqueira Ó Inês A. Carrola J. S. Gonçalves C. (2025). Association between gut microbiota and pediatric obesity: A systematic review. *Nutrition* 140:112875. 10.1016/j.nut.2025.112875 40714355

[B62] Ossom Williamson P. Minter C. I. J. (2019). Exploring PubMed as a reliable resource for scholarly communications services. *J. Med. Libr. Assoc.* 107 16–29. 10.5195/jmla.2019.433 30598645 PMC6300231

[B63] Plovier H. Everard A. Druart C. Depommier C. Van Hul M. Geurts L. et al. (2017). A purified membrane protein from akkermansia muciniphila or the pasteurized bacterium improves metabolism in obese and diabetic mice. *Nat. Med.* 23 107–113. 10.1038/nm.4236 27892954

[B64] Podlesny D. Durdevic M. Paramsothy S. Kaakoush N. O. Högenauer C. Gorkiewicz G. et al. (2022). Identification of clinical and ecological determinants of strain engraftment after fecal microbiota transplantation using metagenomics. *Cell Rep. Med.* 3:100711. 10.1016/j.xcrm.2022.100711 35931074 PMC9418803

[B65] Qin J. Li R. Raes J. Arumugam M. Burgdorf K. S. Manichanh C. et al. (2010). A human gut microbial gene catalogue established by metagenomic sequencing. *Nature* 464 59–65. 10.1038/nature08821 20203603 PMC3779803

[B66] Qin J. Li Y. Cai Z. Li S. Zhu J. Zhang F. et al. (2012). A metagenome-wide association study of gut microbiota in type 2 diabetes. *Nature* 490 55–60. 10.1038/nature11450 23023125

[B67] Quiroga R. Nistal E. Estébanez B. Porras D. Juárez-Fernández M. Martínez-Flórez S. et al. (2020). Exercise training modulates the gut microbiota profile and impairs inflammatory signaling pathways in obese children. *Exp. Mol. Med.* 52 1048–1061. 10.1038/s12276-020-0459-0 32624568 PMC8080668

[B68] Reyes A. Durán C. Rodríguez-Otálora S. Delgado Pugley D. Iraola G. Domínguez-Bello M. G. et al. (2025). A latin American perspective on microbiome research. *Nat. Commun.* 16:10691. 10.1038/s41467-025-66756-y 41298521 PMC12660987

[B69] Ridaura V. K. Faith J. J. Rey F. E. Cheng J. Duncan A. E. Kau A. L. et al. (2013). Gut microbiota from twins discordant for obesity modulate metabolism in mice. *Science* 341:1241214. 10.1126/science.1241214 24009397 PMC3829625

[B70] Rinott E. Youngster I. Meir A. Y. Tsaban G. Kaplan A. Zelicha H. et al. (2021a). Autologous fecal microbiota transplantation can retain the metabolic achievements of dietary interventions. *Eur. J. Intern. Med.* 92 17–23. 10.1016/j.ejim.2021.03.038 33883079

[B71] Rinott E. Youngster I. Yaskolka Meir A. Tsaban G. Zelicha H. Kaplan A. et al. (2021b). Effects of diet-modulated autologous fecal microbiota transplantation on weight regain. *Gastroenterology* 160 158–173.e10. 32860791 10.1053/j.gastro.2020.08.041 PMC7755729

[B72] Ruan Y. Zhu T. Yang R. Su F. An C. Hu Z. et al. (2025). Donor-derived microbial engraftment and gut microbiota shifts associated with weight loss following fecal microbiota transplantation. *Appl. Environ. Microbiol.* 91:e0012025. 10.1128/aem.00120-25 40464558 PMC12285249

[B73] Shaheen N. Khursheed W. Gurung B. Wang S. (2025). *Akkermansia muciniphila*: A key player in gut microbiota-based disease modulationAkkermansia muciniphila. *Microbiol. Res.* 301:128317. 10.1016/j.micres.2025.128317 40845731

[B74] Shin N. R. Lee J. C. Lee H. Y. Kim M. S. Whon T. W. Lee M. S. et al. (2014). An increase in the *Akkermansia spp*. population induced by metformin treatment improves glucose homeostasis in diet-induced obese mice. *Gut* 63 727–735. 10.1136/gutjnl-2012-303839 23804561

[B75] Smits L. P. Kootte R. S. Levin E. Prodan A. Fuentes S. Zoetendal E. G. et al. (2018). Effect of vegan fecal microbiota transplantation on carnitine- and choline-derived trimethylamine-N-oxide production and vascular inflammation in patients with metabolic syndrome. *J. Am. Heart Assoc.* 7:e008342. 10.1161/JAHA.117.008342 29581220 PMC5907601

[B76] Somnuk S. Komindr S. Monkhai S. Poolsawat T. Nakphaichit M. Wanikorn B. (2023). Metabolic and inflammatory profiles, gut microbiota and lifestyle factors in overweight and normal weight young thai adults. *PLoS One* 18:e0288286. 10.1371/journal.pone.0288286 37450433 PMC10348517

[B77] Stoian D. Pescari D. Bena A. Paul C. Mihuta S. (2026). Integrating TMAO into the pathogenesis of obesity and type 2 diabetes: A mini review. *Front. Clin. Diab. Healthc.* 7:1765794. 10.3389/fcdhc.2026.1765794 41685376 PMC12890643

[B78] Synnestvedt M. B. Chen C. Holmes J. H. (2005). CiteSpace II: Visualization and knowledge discovery in bibliographic databases. *AMIA Annu. Symp. Proc.* 2005 724–728. 16779135 PMC1560567

[B79] Tremaroli V. Bäckhed F. (2012). Functional interactions between the gut microbiota and host metabolism. *Nature* 489 242–249. 10.1038/nature11552 22972297

[B80] Turnbaugh P. J. Ley R. E. Mahowald M. A. Magrini V. Mardis E. R. Gordon J. I. (2006). An obesity-associated gut microbiome with increased capacity for energy harvest. *Nature* 444 1027–1031. 10.1038/nature05414 17183312

[B81] Valderrama B. Calderón-Romero P. Bastiaanssen T. F. S. Lavelle A. Clarke G. Cryan J. F. (2025). The south American MicroBiome archive (saMBA): Enriching the microbiome field by studying neglected populations. *Nat. Commun.* 16:7371. 10.1038/s41467-025-62601-4 40783495 PMC12335589

[B82] van Eck N. J. Waltman L. (2010). Software survey: VOSviewer, a computer program for bibliometric mapping. *Scientometrics* 84 523–538. 10.1007/s11192-009-0146-3 20585380 PMC2883932

[B83] van Nood E. Vrieze A. Nieuwdorp M. Fuentes S. Zoetendal E. G. de Vos W. M. et al. (2013). Duodenal infusion of donor feces for recurrent clostridium difficile. *New Engl. J. Med.* 368 407–415. 10.1056/NEJMoa1205037 23323867

[B84] Vishwakarma R. K. Gautam P. Sahu M. Nath G. Yadav B. S. (2025). Gut microbiome in obesity: A narrative review of mechanisms, interventions, and future directions. *Probiot. Antimicrob. Proteins* 10.1007/s12602-025-10855-1 41313537

[B85] Vrieze A. Van Nood E. Holleman F. Salojärvi J. Kootte R. S. Bartelsman J. F. W. M. et al. (2012). Transfer of intestinal microbiota from lean donors increases insulin sensitivity in individuals with metabolic syndrome. *Gastroenterology* 143 913–916.e7. 22728514 10.1053/j.gastro.2012.06.031

[B86] Wexler A. G. Goodman A. L. (2017). An insider’s perspective: Bacteroides as a window into the microbiome. *Nat. Microbiol.* 2:17026. 10.1038/nmicrobiol.2017.26 28440278 PMC5679392

[B87] Wilson B. C. Vatanen T. Jayasinghe T. N. Leong K. S. W. Derraik J. G. B. Albert B. B. et al. (2021). Strain engraftment competition and functional augmentation in a multi-donor fecal microbiota transplantation trial for obesity. *Microbiome* 9:107. 10.1186/s40168-021-01060-7 33985595 PMC8120839

[B88] Wu G. D. Chen J. Hoffmann C. Bittinger K. Chen Y. Y. Keilbaugh S. A. et al. (2011). Linking long-term dietary patterns with gut microbial enterotypes. *Science* 334 105–108. 10.1126/science.1208344 21885731 PMC3368382

[B89] Xu Y. Wang N. Tan H.-Y. Li S. Zhang C. Feng Y. (2020). Function of *Akkermansia muciniphila* in obesity: Interactions with lipid metabolism, immune response and gut systems. *Front. Microbiol.* 11:219. 10.3389/fmicb.2020.00219 32153527 PMC7046546

[B90] Yang J. Li G. Wang S. He M. Dong S. Wang T. et al. (2025). Butyrate prevents obesity accompanied by HDAC9-mediated browning of white adipose tissue. *Biomedicines* 13:260. 10.3390/biomedicines13020260 40002674 PMC11852213

[B91] Youngster I. Mahabamunuge J. Systrom H. K. Sauk J. Khalili H. Levin J. et al. (2016). Oral, frozen fecal microbiota transplant (FMT) capsules for recurrent clostridium difficile infection. *BMC Med.* 14:134. 10.1186/s12916-016-0680-9 27609178 PMC5016994

[B92] Yu E. W. Gao L. Stastka P. Cheney M. C. Mahabamunuge J. Torres Soto M. et al. (2020). Fecal microbiota transplantation for the improvement of metabolism in obesity: The FMT-TRIM double-blind placebo-controlled pilot trial. *PLoS Med.* 17:e1003051. 10.1371/journal.pmed.1003051 32150549 PMC7062239

[B93] Yu H. Zhang Y. Yang D. Luo H. Zhou Y. (2025). Advances in capsule-based fecal microbiota transplantation: Clinical applications and innovations. *J. Transl. Med.* 23:1370. 10.1186/s12967-025-07407-0 41331614 PMC12673741

[B94] Zeevi D. Korem T. Zmora N. Israeli D. Rothschild D. Weinberger A. et al. (2015). Personalized nutrition by prediction of glycemic responses. *Cell* 163 1079–1094. 10.1016/j.cell.2015.11.001 26590418

[B95] Zeng N. Sun J.-X. Liu C.-Q. Xu J.-Z. An Y. Xu M.-Y. et al. (2024). Knowledge mapping of application of image-guided surgery in prostate cancer: A bibliometric analysis (2013–2023). *Int. J. Surg.* 110 2992–3007. 10.1097/JS9.0000000000001232 38445538 PMC11093506

[B96] Zhang F. Zuo T. Wan Y. Xu Z. Cheung C. Li A. Y. et al. (2022). Multi-omic analyses identify mucosa bacteria and fecal metabolites associated with weight loss after fecal microbiota transplantation. *Innovation* 3:100304. 10.1016/j.xinn.2022.100304 36091491 PMC9460156

[B97] Zhang Y. Cao J. Wang Y. Fan X. Deng R. Mi J. (2025). Effects of fecal microbiota transplantation on glycemic and lipid profiles in overweight or obese patients with metabolic disorders: A systematic review and meta-analysis. *Front. Endocrinol.* 16:1737543. 10.3389/fendo.2025.1737543 41473248 PMC12745229

[B98] Zhang Z. Mocanu V. Deehan E. C. Hotte N. Zhu Y. Wei S. et al. (2024). Recipient microbiome-related features predicting metabolic improvement following fecal microbiota transplantation in adults with severe obesity and metabolic syndrome: A secondary analysis of a phase 2 clinical trial. *Gut Microbes* 16:2345134. 10.1080/19490976.2024.2345134 38685731 PMC11062372

[B99] Zhernakova A. Kurilshikov A. Bonder M. J. Tigchelaar E. F. Schirmer M. Vatanen T. et al. (2016). Population-based metagenomics analysis reveals markers for gut microbiome composition and diversity. *Science* 352 565–569. 10.1126/science.aad3369 27126040 PMC5240844

[B100] Zuppi M. Vatanen T. Wilson B. C. Golovina E. Portlock T. Cutfield W. S. et al. (2024). Fecal microbiota transplantation alters gut phage communities in a clinical trial for obesity. *Microbiome* 12:122. 10.1186/s40168-024-01833-w 38970126 PMC11227244

