# Supplementary material for: Fecal microbiota transplantation in obesity: a comprehensive overview from basic research to clinical application
Source: Front Microbiol. 2026 Jun 10;17:1840517. doi: 10.3389/fmicb.2026.1840517 (PMC13291017; doi:10.3389/fmicb.2026.1840517)
Supplement: Supplementary file 1 [file Data_Sheet_1.pdf]

Online Supplementary File

Table S1 Search strategy in the Web of Science

	Search query	Result
#1	TS= (“fecal microbiota transplantation” OR “fecal transplantation” OR “microbiota transplantation” OR “washed microbiota transplantation” OR “faecal transplantation” OR “stool transplants” OR “faecal bacteriotherapy” OR “fecal flora reconstitution” OR “fecal microbiota reconstitution” OR “intestinal microbiota transplantation” OR “fecal microbiota transfusion” OR “stool microbiota transplantation” OR “stool microbiota transfusion”)	7,473
#2	TS= (“obesity” OR “adiposity” OR “adipositas” OR “overweight”)	501,909
#3	#1 And #2	734

Table S2 Summary of date source and selection in Web of Science

Data source	Web of Science
Category	Specific Standard Requirements
Research database	Web of Science Core Collection
Citation indexes	SCI, SSCI
Language	“English”
Searching keywords	“Fecal microbiota transplantation” And “obesity”
Document types	“Articles”, “Review”

Date extraction	Export with full records and cited references in plain text format
Sample	734

Table S3 The search strategy on PubMed

	Search query	Result
#1	All fields = (“fecal microbiota transplantation” OR “fecal transplantation” OR “microbiota transplantation” OR “washed microbiota transplantation” OR “faecal transplantation” OR “stool transplants” OR “faecal bacteriotherapy” OR “fecal flora reconstitution” OR “fecal microbiota reconstitution” OR “intestinal microbiota transplantation” OR “fecal microbiota transfusion” OR “stool microbiota transplantation” OR “stool microbiota transfusion”)	11,252
#2	All fields = (“obesity” OR “adiposity” OR “adipositas” OR “overweight”)	529,767
#3	#2 and #3	737
#4	Article type: clinical trial	30

Table S4 The top 10 countries and institutions on research of application of FMT in obesity

Rank	Country	Counts	Institution	Country	Counts
1	China	246	Zhejiang University	China	18

2	USA	93	Chinese Academy of Sciences	China	15
3	Netherlands	32	University of Amsterdam	Netherlands	15
4	Italy	31	Harvard Medical School	USA	13
5	Sweden	25	University of Gothenburg	Sweden	13
6	Canada	23	China Agricultural University	China	12
7	Spain	23	Hunan Agricultural University	China	12
8	France	22	Southern Medical University	China	12
9	Germany	19	University of Copenhagen	Denmark	12
10	Japan	16	Shanghai Jiao Tong University	China	10

Table S5 The top 10 authors and co-cited Authors on research of application of FMT in obesity

Rank	Authors	Counts	Citations	Co-Cited Authors	Citations
1	Nieuwdorp M	19	2061	Cani PD	377

2	Wang Jing	9	538	Turnbaugh PJ	371
3	Koren O	7	275	Bäckhed F	225
4	Wang Pan	6	589	Ley RE	216
5	Yin Yulong	6	415	Vrieze A	171
6	Chen Fang	5	581	Everard A	147
7	Cutfield WS	5	88	Ridaura VK	133
8	Gasbarrini A	5	130	Qin Junjie	124
9	Herrema H	5	335	Kootte JS	83
10	Hu Xiaosong	5	581	Bajaj JS	77

Table S6 The top 10 journals and co-cited journals of FMT in obesity

Rank	Journals	IF	Counts	Co-Cited Academic Journals	IF	Counts
1	Gut Microbes	10.931	21	Nature	47.840	1708

2	Frontiers in Microbiology	4.504	19	Gut	25.503	1136
3	Nutrients	4.919	14	Gastroenterology	25.214	954
4	Gut	25.503	12	Proceedings of the National Academy of Sciences of the United States of Am*erica*	8.872	933
5	Microbiome	12.683	9	Science	44.692	885
6	Microorganisms	4.149	9	Plos One	2.571	881
7	Scientific Reports	3.899	9	Nutrients	4.019	823
8	Food & Function	5.413	8	Cell Metabolism	30.755	729
9	Frontiers in Endocrinology	4.566	8	Scientific Reports	3.899	665
10	Frontiers in Cellular and Infection Microbiology	4.746	7	Cell	42.256	640

Table S7 The detailed analysis information of keyword in different clusters

CITATION COUNTS			BURSTS			DEGREE			CENTRALITY			SIGMA		
Citation	Node Name	Cluster	Bursts	Node Name	Cluster	Degree	Node Name	Cluster	Centrality	Node Name	Cluster	Sigma	Node Name	Cluster

Counts		ID			ID			ID			ID			ID
280	gut microbiota	7	6.99	intestinal microbiota	8	21	diversity	8	0.18	diversity	8	2.62	*Clostridium difficile* infection	5
177	obesity	8	5.74	*Clostridium difficile* infection	5	19	high fat diet	2	0.18	*Clostridium difficile* infection	5	1.85	fecal transplantat ion	4
146	intestinal microbiota	8	5.41	Diet-induce d-obesity	6	19	disease	5	0.16	fecal transplantat ion	4	1.54	inflammato ry bowel disease	11
131	fecal microbiota transplantation	9	5.15	Inflammato ry bowel disease	11	17	*Clostridium difficile* infection	5	0.16	dietary fiber	11	1.51	diversity	8
91	insulin resistance	4	4.38	fecal microbiota	1	17	adipose tissue	3	0.15	adipose tissue	3	1.41	fecal microbiota	1
88	metabolic syndrome	2	4.09	fecal transplantat ion	4	17	bacteria	8	0.14	high fat diet	2	1.38	glucagon like peptide 1	4
86	inflammation	9	3.48	risk	13	16	activation	0	0.14	disease	5	1.28	high fat diet	2
64	diet induced obesity	6	3.48	bacteriother apy	8	16	diet	8	0.14	impact	6	1.25	activation	0
63	gut microbiome	8	3.28	chain fatty acids	0	15	glucagon like peptide 1	4	0.13	bacteria	8	1.24	disease	5
63	insulin	4	3.26	*Clostridium*	8	15	association	3	0.13	activation	0	1.22	short-chain	1

	sensitivity			*difficile*									fatty acids	

Bursts: The burst strength of a keyword, indicating a rapid increase in citation frequency over a specific period. Higher values denote stronger research frontiers or emerging hotspots.

Degree: The number of direct co-occurrence connections a keyword has with other keywords, representing its relational strength within the network.

Centrality: A measure (typically between 0 and 1) of the importance of a keyword node in the co-occurrence network. High centrality (e.g., >0.1) suggests the keyword serves as a critical hub bridging different research clusters.

Sigma: A metric that combines betweenness centrality and burst strength to identify novel or pivotal keywords. Higher sigma values indicate a keyword that is both central in the network and exhibits strong citation burst activity, often representing a transformative or emerging research direction.

Table S8 The summary of clinical trials

**Reference**	**Frozen or fresh FMT?**	**Autologous or Allogenic FMT**	**Dietary interventions**	**Exercise intervention**	**Drug delivery**	**Antibiotics or Bowel preparation**	**FMT dose**	**Clinical efficacy**	**Gut Microbial Composition changes following FMT**
Loek P Smits(Smits et al., 2018)	Fresh	Both	N	NG	Duodenal infusion	Y	The complete stool production was diluted with 500 mL of sterile saline (0.9%) and subsequently stirred and filtered, resulting in a 500-mL homogenized	Intestinal microbiome changes+; Fecal microbiota diversity-; Vessel wall inflammation-; TMAO production-.	**Allogenic FMT:**<br>↑: *Lachnospiraceae: Bryantella formatexigens, Megamonas hypermegale, Lachnobacterium bovis*<br><br>**Autologous FMT:**<br>↑: *Alcaligenes faecalis, Akkermansia*

							solution.		
Witjes JJ(Witjes et al., 2020)	Fresh	Both	N	NG	Duodenal infusion	Y (no antibiotics)	The complete stool production was diluted with 500 mL of sterile saline (0.9%) and subsequently stirred and filtered, resulting in a 500-mL homogenized solution. Both were performed three times at 8-week intervals.	Plasma metabolites and fecal microbiota composition+; Fecal microbiota diversity-; Histological and liver gene expression+.	**Allogenic FMT:**<br>↑: *Ruminococcus, Eubacterium hallii, Faecalibacterium, Prevotella copri*<br><br>**Autologous FMT:**<br>↑: *Lachnospiraceae*
Jessica R Allegretti(Allegretti et al., 2020)	NG	Allogenic	N	NG	Oral capsules	N	The mean stool per capsule was 0.75 gm with 30-capsule single induction dose and a maintenance dose of 12 capsules at week 4 and week 8.	Weight loss -; Metabolic effects +	↑: *Faecalibacterium*
Elaine W Yu(Ew et al., 2020)	Frozen	Allogenic	N	N	Oral capsules	N	Participants were administered 15 capsules derived	Metabolic effects -; Gut microbiota engraftment +;	↑: *Prevotella*

							from approximately 24 grams of fecal material on each of 2 consecutive days at week 0, followed by 15 capsules once a week for the next 5 weeks.	Microbiome diversity-.	
Karen S W Leong(Ksw et al., 2020)	Frozen	Allogenic	N	N	Oral capsules	Y	Single course of oral encapsulated fecal microbiome. Participants received 28 capsules, which equated to approximately 22 g (wet weight) of fecal material (approximately 14 mL of frozen microbial suspension or saline) over 2 consecutive days.	Weight loss-; Gut microbial diversity or composition-	↓: *Escherichia coli*<br>↑: *Faecalibacterium prausnitzii, Alistipes spp.*
Annick V Hartstra(Hartstra et	Fresh	Both	N	NG	Filtrate via gastroscopy	Y	Fresh morning stools were diluted in 500 mL of 0.9%	Potential modulation of brain dopamine	↑: *Bacteroides uniformis, Faecalibacterium prausnitzii, Alistipes spp., Bacteroides*

al., 2020)							saline used for the FMT.	and serotonin transporters.	*ovatus, Bacteroidales bacterium ph8*<br>↓: *Prevotella spp. / Prevotella copri, Escherichia coli*
Laura Craven(Craven et al., 2020)	Fresh	Both	NO	NG	Endoscope	Y	2 g of stool with 125 mL of sterile saline.	Metabolic effects -	↑: *Bacteroides spp., Faecalibacterium prausnitzii, Alistipes spp., Lactobacillaceae, Bacteroidales bacterium ph8*<br>↓: *Prevotella spp., Escherichia coli,*<br>*Ruminococcaceae* UCG-005
Rinott E(Rinott et al., 2021b)	Frozen	Autologous	Y	Y	Oral capsules	NG	10 grams of aFMT capsules delivered 10 times (a total of ~100g fecal matter) over a 6-month period. Administration sessions were held weekly for the first month, and every 3 weeks thereafter for a total of 10 sessions.	Weight loss+; glycemic control+; intestinal microbiome changes (green-Mediterranean diet).	**Mediterranean group**:<br>↑: *Akkermansia muciniphila*<br>↓: *Lactobacillus ruminis*<br><br>**Autologous FMT in Mediterranean group**:<br>↑: *Roseburia hominis*<br><br>**Green Mediterranean group:**<br>↑: *Bacteroides massiliensis, Paraprevotella clara*<br><br>**Autologous FMT in the green Mediterranean:**<br>↑ : *Alistipes putredinis,*

									Bacteroides vulgatus, *Bacteroides uniformis*.
Mocanu V(V et al., 2021)	Frozen	Allogenic	Y	NG	oral FMT capsules	Y	A single dose of 20 FMT capsules (weighing 50g in total).	Insulin sensitivity improvement+ (FMT-LF); Intestinal microbiome changes+.	**FMT-LF**<br>↑: *Phascolarcobacterium*, *Christensenellaceae*, *Bacteroides*, *Akkermansia muciniphila*<br>↓: *Dialister*, *Ruminococcus torques*
Rinott E(Rinott et al., 2021a)	Frozen	Autologous	Y	Y	Oral capsules	NG	10 grams of aFMT capsules delivered 10 times (a total of ~100g fecal matter) over a 6-month period.	Preservatory effect on gut microbiome composition; beneficial cardiometabolic effects.	**Mediterranean group:**<br>↑: *Akkermansia muciniphila*;<br>↓: *Lactobacillus ruminis*<br><br>**Autologous FMT in Mediterranean group:**<br>↑: *Roseburia hominis*<br><br>**Green Mediterranean group:**<br>↑: *Bacteroides massiliensis, Paraprevotella clara*<br><br>**Autologous FMT in the green Mediterranean:**<br>↑ : *Alistipes putredinis, Bacteroides vulgatus, Bacteroides uniformis*

Wilson BC(Wilson et al., 2021)	Frozen	Allogenic	N	N	Oral capsules	Y	A total of 28 capsules containing 7g of concentrated fecal microbiota over two consecutive days.	Gut microbiota engraftment (a high P/B ratio).	FMT: P/B ratio ↑<br><br>In females: *Megamonas hypermegale*, *Megamonas rupellensis*, *Bacteroides finegoldii*, *Bacteroides salyersiae*, *Bacteroides faecis*, *Bacteroides massiliensis*, *Prevotella copri*, *Desulfovibrio piger*, *Barnesiella intestinihominis*<br><br>In males: *Catenibacterium mitsuokai*, *Bacteroides finegoldii*, *Prevotella copri, Collinsella aerofaciens*, *Ruminococcus lactaris*
Lanfeng Xue(Xue et al., 2022)	Fresh	Allogenic	Y	Y	Colonoscopy followed by enemas	NG	Patients received a total of 200 ml of fresh bacteria solution per day, for 3 days in total.	Gut microbiota abundance +; Intestinal microbiome changes+.	↑: *Bacteroides, Paraprevotella*<br>↓: *Escherichia-Shigella*
Siew C Ng(Ng et al., 2022)	Frozen	Allogenic	Y?	NG	OGD	NG	100–200 mL of FMT solution with 50 g stool over 2–3 min into the distal	Gut microbiota engraftment+; Metabolic effects and Intestinal	**FMT:**<br>↑: *Prevotella copri* and several butyrate-producing bacteria: *Faecalibacterium prausnitzii,*

							duodenum every 4 weeks for up to week 12.	microbiome changes+ (FMT plus LSI group).	*Collinsella tanakaei, Anaerostipes hadrus, several Eubacterium spp.* and *Coprococcus spp.*<br>↓: *Clostridium clostridioforme*; *Fusobacterium ulcerans*<br><br>**FMT plus LSI:**<br>↑: *Bifidobacterium spp.* and *Lactobacillus spp.*
Lahtinen P(Lahtinen et al., 2022)	Frozen	Both	Y	NG	Gastroscopy	Y	A frozen and thawed solution of donor feces (30 g in a 170 mL final volume of saline and 10% glycerol).	Weight loss-.	NG
Fen Zhang(Zhang et al., 2022)	NG	Allogenic	N	NG	OGD	Y	Dose: NG.<br>Frequency: All of them have received 4-week FMT infusions by OGD (1st day and 3rd day each week) and enema (2nd, 4th and 5th each week).	a minor body weight loss; individualized response of fecal and mucosa microbiome+.	↑: *Bacteroides genus*, *Bifidobacterium*, *Bacteriodes vulgatus* and *Alistipes onderdonki*
Yasaman	Fresh	Both	NG	NG	Colonosco	Y	A single-dose FMT	Intestinal	**1 month:**

Ghorbani(Ghorbani et al., 2023)					py		with 500 mL fecal filtrate (50 grams of donated feces) was infused into the caecum via a colonoscope.	microbiome changes+; Metabolites and metagenomic pathway +; HOMA-IR and clinical variables-.	**Allogenic group:**<br>↑: *Bacteroides xylanisolvens*, *Lactococcus lactis*<br>↓: *Escherichia coli*<br><br>**Autologous group:**<br>↓: *Blautia obeum*, *Flavonifractor plautii*<br><br>**3 months:**<br>**Allogenic group**<br>↓: *Roseburia intestinalis*<br><br>**Autologous group**<br>↓: *Roseburia hominis*
Kamer O(Kamer et al., 2023)	Frozen	Autologous	Y	Y	Oral capsules	NG	10 grams of aFMT capsules delivered 10 times (a total of ~100g fecal matter) over a 6-month period.	Weight loss maintenance+ (low-abundance taxa).	NG
Michele Zuppi(Zuppi et al., 2024)	Frozen	Allogenic	N	N	Oral FMT capsules	Y	28 capsules (approximately 14 mL of frozen microbial suspension)	Overall microbial populations and phage changes+.	FMT increased the variability and diversity of recipient gut phageomes.

							administered over two consecutive mornings, specifically 16 capsules in the first morning and 12 capsules in the second morning.		
Zhengxiao Zhang(Zhang et al., 2024)	Frozen	Allogenic	Y	NG	Oral FMT capsules	Y	A single dose of FMT (50 g of donor stool) with 20 capsules.	Recipients' factors at baseline (microbiota engraftment); Specific donor-specific microbes.	Responders：<br>↑: *Faecalibacillus intestinalis* (ASV44), *Roseburia spp*. (ASV103), and *Christensenellaceae spp.* (ASV140)
Wilson BC(Wilson et al., 2025)	Frozen	Allogenic	N	N	Oral capsules	Y	A total of 28 capsules containing 7g of concentrated fecal microbiota over two consecutive days.	Body composition and metabolic health+; Gut microbiome richness, composition and functional capacity+.	↑: *Alistipes sp.* CAG 435, *Bacteroides finegoldii*, *Bacteroides salyersiae*, and *Desulfovibrionaceae bacterium*<br><br>Phageome：<br>↑: *Myoviridae*<br>↓: *Siphoviridae*
Yuting Ruan(Ruan et al.,	Fresh	Allogenic	N	N	Nasojejunal tube		FMT was performed biweekly for three sessions using 200	Weight loss+; Intestinal microbiome	Responders：<br>↑: *Phascolarctobacterium, Acidaminococcaceae*

2025)							mL of fresh fecal microbiota solution delivered into the duodenum.	changes; Gut microbiota engraftment; Microbiota-targeted therapy (*Phascolarctobacterium* and *Acidaminococcaceae*).	Non-responders: ↑: *Paraprevotella*

N: No; Y: Yes; NG: Not Given; cFMT: Capsule-based fecal microbiota transplantation; OGD: Oesophago-gastro-duodenoscopy; LSI: lifestyle intervention; TMAO: trimethylamine-N-oxide

Reference:

Allegretti, J. R., Kassam, Z., Mullish, B. H., Chiang, A., Carrellas, M., Hurtado, J., et al. (2020). Effects of fecal microbiota transplantation with oral capsules in obese patients. *Clin. Gastroenterol. Hepatol. Off. Clin. Pract. J. Am. Gastroenterol. Assoc.* 18, 855-863.e2. doi: 10.1016/j.cgh.2019.07.006

Craven, L., Rahman, A., Nair Parvathy, S., Beaton, M., Silverman, J., Qumosani, K., et al. (2020). Allogenic fecal microbiota transplantation in patients with nonalcoholic fatty liver disease improves abnormal small intestinal permeability: A randomized control trial. *Am. J. Gastroenterol.* 115, 1055–1065. doi: 10.14309/ajg.0000000000000661

Ew, Y., L, G., P, S., Mc, C., J, M., M, T. S., et al. (2020). Fecal microbiota transplantation for the improvement of metabolism in obesity: The FMT-TRIM double-blind placebo-controlled pilot trial. *PLoS Med.* 17. doi: 10.1371/journal.pmed.1003051

Ghorbani, Y., Schwenger, K. J. P., Sharma, D., Jung, H., Yadav, J., Xu, W., et al. (2023). Effect of faecal microbial transplant via colonoscopy in patients with severe obesity and insulin resistance: A randomized double-blind, placebo-controlled phase 2 trial. *Diabetes Obes. Metab.* 25, 479–490. doi: 10.1111/dom.14891

Hartstra, A. V., Schüppel, V., Imangaliyev, S., Schrantee, A., Prodan, A., Collard, D., et al. (2020). Infusion of donor feces affects the gut-brain axis in humans with metabolic syndrome. *Mol. Metab.* 42, 101076. doi: 10.1016/j.molmet.2020.101076

Kamer, O., Rinott, E., Tsaban, G., Kaplan, A., Yaskolka Meir, A., Zelicha, H., et al. (2023). Successful weight regain attenuation by autologous fecal microbiota transplantation is associated with non-core gut microbiota changes during weight loss; randomized controlled trial. *Gut Microbes* 15, 2264457. doi:

10.1080/19490976.2023.2264457

Ksw, L., Tn, J., Bc, W., Jgb, D., Bb, A., V, C., et al. (2020). Effects of fecal microbiome transfer in adolescents with obesity: The gut bugs randomized controlled trial. *JAMA Netw. Open* 3. doi: 10.1001/jamanetworkopen.2020.30415

Lahtinen, P., Juuti, A., Luostarinen, M., Niskanen, L., Liukkonen, T., Tillonen, J., et al. (2022). Effectiveness of fecal microbiota transplantation for weight loss in patients with obesity undergoing bariatric surgery: A randomized clinical trial. *JAMA Netw. Open* 5, e2247226. doi: 10.1001/jamanetworkopen.2022.47226

Ng, S. C., Xu, Z., Mak, J. W. Y., Yang, K., Liu, Q., Zuo, T., et al. (2022). Microbiota engraftment after faecal microbiota transplantation in obese subjects with type 2 diabetes: A 24-week, double-blind, randomised controlled trial. *Gut* 71, 716–723. doi: 10.1136/gutjnl-2020-323617

Rinott, E., Youngster, I., Meir, A. Y., Tsaban, G., Kaplan, A., Zelicha, H., et al. (2021a). Autologous fecal microbiota transplantation can retain the metabolic achievements of dietary interventions. *Eur. J. Intern. Med.* 92, 17–23. doi: 10.1016/j.ejim.2021.03.038

Rinott, E., Youngster, I., Yaskolka Meir, A., Tsaban, G., Zelicha, H., Kaplan, A., et al. (2021b). Effects of diet-modulated autologous fecal microbiota transplantation on weight regain. *Gastroenterology* 160, 158-173.e10. doi: 10.1053/j.gastro.2020.08.041

Ruan, Y., Zhu, T., Yang, R., Su, F., An, C., Hu, Z., et al. (2025). Donor-derived microbial engraftment and gut microbiota shifts associated with weight loss following fecal microbiota transplantation. *Appl. Environ. Microbiol.* 91, e0012025. doi: 10.1128/aem.00120-25

Smits, L. P., Kootte, R. S., Levin, E., Prodan, A., Fuentes, S., Zoetendal, E. G., et al. (2018). Effect of vegan fecal microbiota transplantation on carnitine- and choline-derived trimethylamine-N-oxide production and vascular inflammation in patients with metabolic syndrome. *J. Am. Heart Assoc. Cardiovasc. Cerebrovasc. Dis.* 7, e008342. doi: 10.1161/JAHA.117.008342

V, M., Z, Z., Ec, D., Dh, K., N, H., S, K., et al. (2021). Fecal microbial transplantation and fiber supplementation in patients with severe obesity and metabolic syndrome: A randomized double-blind, placebo-controlled phase 2 trial. *Nat. Med.* 27. doi: 10.1038/s41591-021-01399-2

Wilson, B. C., Vatanen, T., Jayasinghe, T. N., Leong, K. S. W., Derraik, J. G. B., Albert, B. B., et al. (2021). Strain engraftment competition and functional augmentation in a multi-donor fecal microbiota transplantation trial for obesity. *Microbiome* 9, 107. doi: 10.1186/s40168-021-01060-7

Wilson, B. C., Zuppi, M., Derraik, J. G. B., Albert, B. B., Tweedie-Cullen, R. Y., Leong, K. S. W., et al. (2025). Long-term health outcomes in adolescents with obesity treated with faecal microbiota transplantation: 4-year follow-up. *Nat. Commun.* 16, 7786. doi: 10.1038/s41467-025-62752-4

Witjes, J. J., Smits, L. P., Pekmez, C. T., Prodan, A., Meijnikman, A. S., Troelstra, M. A., et al. (2020). Donor fecal microbiota transplantation alters gut microbiota and metabolites in obese individuals with steatohepatitis. *Hepatol. Commun.* 4, 1578–1590. doi: 10.1002/hep4.1601

Xue, L., Deng, Z., Luo, W., He, X., and Chen, Y. (2022). Effect of fecal microbiota transplantation on non-alcoholic fatty liver disease: A randomized clinical trial. *Front. Cell. Infect. Microbiol.* 12, 759306. doi: 10.3389/fcimb.2022.759306

Zhang, F., Zuo, T., Wan, Y., Xu, Z., Cheung, C., Li, A. Y., et al. (2022). Multi-omic analyses identify mucosa bacteria and fecal metabolites associated with weight loss after fecal microbiota transplantation. *The Innovation* 3, 100304. doi: 10.1016/j.xinn.2022.100304

Zhang, Z., Mocanu, V., Deehan, E. C., Hotte, N., Zhu, Y., Wei, S., et al. (2024). Recipient microbiome-related features predicting metabolic improvement following fecal microbiota transplantation in adults with severe obesity and metabolic syndrome: A secondary analysis of a phase 2 clinical trial. *Gut Microbes* 16, 2345134. doi: 10.1080/19490976.2024.2345134

Zuppi, M., Vatanen, T., Wilson, B. C., Golovina, E., Portlock, T., Cutfield, W. S., et al. (2024). Fecal microbiota transplantation alters gut phage communities in a clinical trial for obesity. *Microbiome* 12, 122. doi: 10.1186/s40168-024-01833-w
